# Neuronal calcium spikes enable vector inversion in the *Drosophila* brain

**DOI:** 10.1101/2023.11.24.568537

**Published:** 2023-11-28

**Authors:** Itzel G. Ishida, Sachin Sethi, Thomas L. Mohren, L.F. Abbott, Gaby Maimon

**Affiliations:** 1 Laboratory of Integrative Brain Function and Howard Hughes Medical Institute, The Rockefeller University, New York NY, USA; 2 Mortimer B. Zuckerman Mind Brain Behavior Institute, Department of Neuroscience, Columbia University, New York NY, USA

## Abstract

A typical neuron signals to downstream cells when it is depolarized and firing sodium spikes. Some neurons, however, also fire calcium spikes when hyperpolarized. The function of such bidirectional signaling remains unclear in most circuits. Here we show how a neuron class that participates in vector computation in the fly central complex employs hyperpolarization-elicited calcium spikes to invert two-dimensional mathematical vectors. When cells switch from firing sodium to calcium spikes, this leads to a ~180° realignment between the vector encoded in the neuronal population and the fly’s internal heading signal, thus inverting the vector. We show that the calcium spikes rely on the T-type calcium channel Ca-α1T, and argue, via analytical and experimental approaches, that these spikes enable vector computations in portions of angular space that would otherwise be inaccessible. These results reveal a seamless interaction between molecular, cellular and circuit properties for implementing vector math in the brain.

## Introduction

Neural circuitry that implements vector mathematics has been characterized in the insect central complex^1–4^. The output of one such circuit in *Drosophila* is a calcium signal––evident across a genetically defined neuronal population––that tracks the fly’s traveling direction referenced to external cues, that is, in allocentric coordinates^1,5^. This signal is constructed from the activity of four presynaptic neuronal populations, each of which can be modeled as encoding a two-dimensional mathematical vector^1^. The lengths of these neuronally encoded vectors are controlled by the fly’s traveling direction referenced to its body axis––i.e., the egocentric traveling direction––and their angles are controlled by the fly’s allocentric heading angle. A downstream neuronal population functionally sums the four vectors to build an estimate of the fly’s allocentric traveling direction^1^.

Formally, this computation could be implemented by summing only two vectors––akin to x and y components in Cartesian geometry––provided that each component vector could point in either the positive or negative direction along its respective axis. The traveling-direction circuit uses four vectors because the neuronal populations encoding each vector can only signal to downstream cells when their constituent neurons are depolarized. This rectification means that only one projection direction along an axis can be signaled and thus two vectors are needed to cover both the positive and negative directions.

We wondered whether a single neuronal population in the *Drosophila* central complex might be able to encode a vector that is invertible, that is, a vector that can point in either of two directions offset by 180°. We found that a specific class of neurons, PFNa cells, can indeed do this by virtue of single PFNa cells expressing two classes of action potentials: sodium spikes induced by membrane depolarization and calcium spikes induced by membrane hyperpolarization. Sodium spikes allow the PFNa population to signal vectors pointing along one axis direction and calcium spikes allow the same population to signal vectors pointing along the inverse direction. We further found that the hyperpolarization-elicited calcium spikes in PFNa cells are mediated by T-type calcium channels, which are known for producing analogous calcium spikes and burst firing in mammalian thalamocortical circuits^6,7^. Finally, we found that neurons monosynaptically downstream of PFNa cells can perform a vector-addition-like read out of the two PFNa populations when both vectors are optogenetically made to have equal lengths and point in either the sodium-spike encoded directions or the inverse, calcium-spike encoded directions. These results––supported by a model rooted in detailed anatomy and physiology––reveal a mechanism by which a central-brain circuit can circumvent canonical rectification constraints on neuronal signaling to implement invertible, two-dimensional vectors.

## Results

### How neuronal populations could encode invertible, two-dimensional vectors

**Figure 1.**
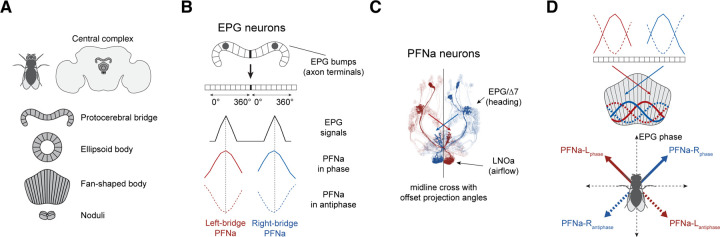
A framework for implementing vector inversions in the *Drosophila* central complex. **(A)** Schematic of the *Drosophila* brain and the central complex. **(B)** EPG neurons tile the ellipsoid body with their dendrites and the protocerebral bridge with their axons. In the ellipsoid body, the EPG dendrites express a single bump of calcium activity whose position around the structure tracks the fly’s heading. Due to anatomy, one copy of the heading bump exists in the EPG axons of the left protocerebral bridge, and a second copy exists in the right bridge (top, black dots and curves). The two copies of the EPG bump slide along the bridge when the fly’s heading changes. EPG neurons drive activity in varying downstream columnar cells whose dendrites tile the bridge, including PFNa neurons (middle, red, and blue traces). The shape of the two PFNa bumps is expected to be sinusoidal, with their phase (i.e., their position in the bridge) and amplitude representing the angle and length of two vectors, in a so-called phasor representation^1,3,5^. If the two peaks of the PFNa sinusoids could invert (i.e. rotate 180°), then this would be equivalent to inverting the vector they encode (bottom, red and blue dotted traces). **(C)** Left- and right-bridge PFNa neurons project to the fan-shaped body with anatomical offsets that introduce, approximately, ±45°^4^ rotations in the angles of the two vectors they encode. Those PFNa neurons that receive EPG (heading) input in the left bridge also receive LNOa input in the right nodulus, i.e., on the opposite side of the midline. The nodulus inputs to PFNa neurons are tuned to the direction of airflow experienced by the fly^12^. **(D)** Graphical representation of how the two sinusoidal activity bumps in the PFNa neurons of the protocerebral bridge are transmitted to the fan-shaped body. In the bridge, the left- and right-bridge PFNa sinusoids are phase aligned to the EPG signal on each side of the bridge (red and blue solid curves at the top) and these sinusoids are offset by ~±45°, due to the PFNa anatomy, when they get transmitted to the fan-shaped body (red and blue solid curves inside the fan-shaped body schematic). As a result, the two vectors that these PFNa phasors represent are offset by ~±45° in the fan-shaped body (red and blue solid vectors in diagram at the bottom). If either PFNa sinusoid in the bridge were to invert its phase (red and blue dotted curves at the top) then this would invert the direction of the encoded vector (red and blue dotted vectors in diagram at the bottom).

Spatial vector computations in the fly central complex (Figure 1A) are anchored to a common, internally generated sense of heading signaled by EPG cells^8,9^. Individual EPG cells innervate one wedge of the circular ellipsoid body with their dendrites and one glomerulus of the linear protocerebral bridge with their axons, with the full population of EPG neurons tiling these two structures. The fly’s heading is indicated by a spatially localized calcium signal, or bump, across the EPG population. This bump rotates around the ellipsoid body as the fly turns, with its position around the ellipsoid body, or phase, tracking the fly’s heading. There are two copies of the ellipsoid body bump present in the EPG axons within the protocerebral bridge (Figure 1B). In total, the EPG system thus consists of three neural rings that represent angular space, one closed ring in the ellipsoid body and two open rings in the protocerebral bridge (Figure 1B).

In the bridge, EPG cells provide synaptic input to dozens of downstream neurons, including several classes of PFN cells^4^. Whereas the two EPG calcium bumps in the bridge are often narrow in shape, the downstream pair of bumps in each class of PFN cells––one in the left bridge and one in the right bridge––are broader, conforming well to sinusoidal functions across the bridge^1,4^ (Figure 1B). A sinusoidal reformatting between EPG and PFN bumps makes sense in light of extensive inputs that PFN cells receive from a set of bridge interneurons called Δ7 cells, whose anatomy positions them to act as sinusoidal spatial-convolutional filters^4,10^. The resulting sinusoidal activity profile of each PFN population appears to encode a two-dimensional vector in a so-called phasor representation. The amplitude of the sinusoid encodes the vector’s length and its peak position, or phase, encodes the vector’s angle^1–3,11^.

The phases of the PFN bumps in the left and right bridge are both yoked to the EPG signal and thus, in the bridge, the two PFN-encoded vectors both point in the fly’s heading direction. However, when PFN populations project from the bridge to the fan-shaped body, they do so with a precise anatomical offset that is equivalent to rotating the right-bridge vector ~45° clockwise and the left-bridge vector ~45° counterclockwise^1,4^ (Figures 1C and 1D). The two PFN vectors thus develop a ~90° offset between them in the fan-shaped body. PFN-recipient neurons in the fan-shaped body perform a vector sum across one or more such vector pairs. They do so by adding, column by column, the sinusoidal activity patterns across their input PFN populations^1^, which produces a sinusoidal activity pattern in the recipient neuronal population whose amplitude and phase match the length and angle of the vector sum of the input vectors.

Adding a pair of vectors with a 90° offset allows the output vector to point anywhere between them, that is, within a 90° sector of angular space. Other parts of angular space can only be covered by including a second vector pair^1,3,5^ or by allowing the vectors within the one pair to invert their direction. We thus hypothesized that there might exist situations in which PFN-encoded vectors could invert, such that each vector could align with either the positive or negative direction of its particular axis. Physiologically, when a vector needs to point in the positive direction, one would observe the standard alignment between the peak of the PFN sinusoid and the EPG bump driving it in the bridge (Figures 1B and 1D, solid lines). Conversely, when the vector needs to point in the inverse direction, there would be a ~180° offset between the PFN and EPG signals in the bridge (Figures 1B and 1D, dotted lines). A 180° offset of a phasor is equivalent to inverting the direction of the encoded vector.

### Population calcium signals in PFNa neurons signal invertible vectors

**Figure 2.**
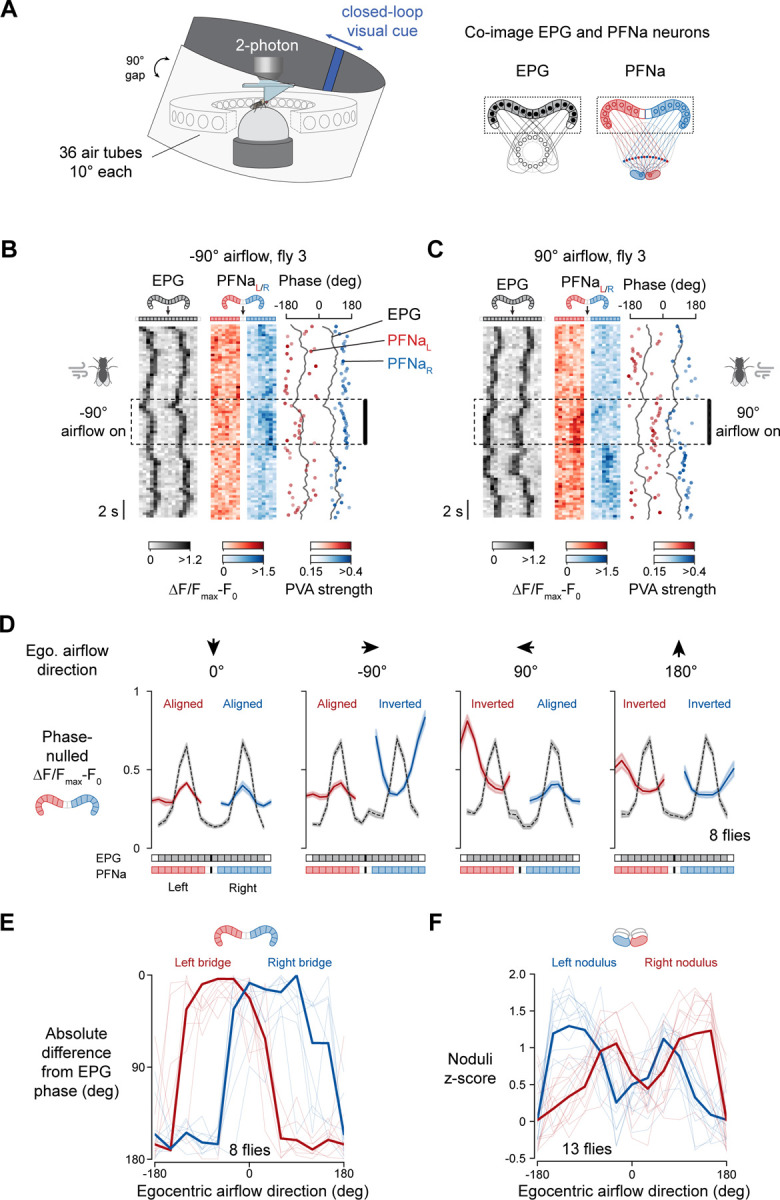
PFNa neurons express activity bumps that can be both in-phase and antiphase relative to the EPG bumps in the protocerebral bridge. **(A)** Imaging neural activity in PFNa neurons in head-fixed flies walking in a virtual reality environment. The flies receive visual feedback in closed loop on their orientation in the environment (blue bar) alongside air puffs, in open loop, from varying directions (see main text and Methods for details). PFNa neurons cross the midline in their anatomical projections from the protocerebral bridge to the fan-shaped-body/nodulus; we always depict right-bridge/left nodulus PFNa neurons in blue and left-bridge/right nodulus neurons in red. **(B)** Simultaneous calcium imaging of EPG and PFNa neurons in the protocerebral bridge. Gray heatmap: jRGECO1a signal from EPG neurons. Red and blue heatmap: GCaMP7f signal from PFNa neurons. The estimated phases of the EPG, left-bridge PFNa, and right-bridge PFNa activity bumps are shown in the third column. The EPG phase on each side of the bridge is shown with a black trace. The PFNa phases are depicted as dots whose opacity scales with the length of the population vector average (PVA) for the relevant bump (i.e., with the size of the bump); data points below PVA strength of 0.15 are not displayed. Air puff time period (4 s) and direction (−90°, from the fly’s left) is indicated. **(C)** Same as B, but for an air-puff stimulus from 90° (from the fly’s right). **(D)** Population-averaged, phase-nulled EPG bumps (gray traces) (Methods). We rotated the PFNa bumps by the same amount as the EPG bumps on each frame, averaged the resultant signals, and plotted the population-averaged PFNa curves over the EPG curves (red and blue traces). We show overlaid curves for four of the twelve air puff angles tested (0°, −90°, +90°, and 180°). **(E)** Absolute difference between the phase of the left- and right-bridge PFNa bumps as a function of the airflow direction. 0° corresponds to perfect alignment of the PFNa phase and the EPG phase on either side of the protocerebral bridge. Thin lines show the averaged data from single flies. Bold lines show the population averages. (**F)** Mean PFNa activity in the noduli as a function of the airflow direction. Thin lines show the averaged data from single flies. Bold lines show the population averages.

Anatomical considerations^4^ suggested to us that PFNa cells––a specific subtype of PFN neuron––might perform vector operations with only two vectors, where each vector is invertible. To study vector computation in PFNa neurons, we placed head-fixed flies on an air-cushioned ball and allowed them to navigate in a simple virtual environment as we imaged neural activity with a two-photon microscope (Figure 2A). The flies’ left/right turns controlled the position of a bright vertical bar on a panoramic visual display, such that the bar’s angular position tracked the fly’s heading in the virtual world akin to a distant cue in the real world, like the sun. Because PFNa neurons have been previously shown to be responsive to airflow stimuli^12^, they seemed poised to transform the direction of airflow from egocentric to allocentric coordinates^12^. Thus, we also surrounded the flies with a ring of 36 static tubes that allowed us to deliver airflow stimuli from various directions (Methods, Figure 2A).

One complication is that airflow can induce the EPG heading bump (i.e., the fly’s sense of allocentric heading) to rotate under certain circumstances^13^. We verified that with our protocol––brief pulses of airflow delivered in open loop from randomized directions––the EPG heading bump did not measurably rotate in response to each puff (Figure S1). In other words, the fly seemed to interpret each air puff as a disturbance arriving from a different allocentric direction rather than its body having turned in the context of a static wind direction. We could thus study how PFNa neurons signal the direction of each puff.

We co-imaged calcium in the axon terminals of EPG cells and in the dendrites of PFNa cells in the protocerebral bridge as flies walked with a closed-loop bar and experienced air puffs from various directions (Methods). The EPG bumps in the bridge were consistently measurable during these experiments. The PFNa bumps, on the other hand, were often dim, but typically became clearer during puffs (Figures 2B and 2C). Notably, when we puffed air on a single fly from the left, the peak of the left-bridge PFNa bump was aligned to the peak of the EPG signal, but the peak of the right-bridge PFNa bump was offset from the EPG peak by ~180° (Figure 2B, dotted box). When we puffed air on the right side of the same fly, the opposite relationship was evident; the left-bridge PFNa bump was now antiphase to the EPG peak (Figure 2C, dotted box). These findings stand in contrast to what is observed in the traveling direction system, where the activity bumps of all four PFN populations always align with the EPG peak in the bridge^1,5^.

For each imaging frame, we computationally shifted the EPG bumps to the same position in the bridge and rotated the PFNa bumps by this same, EPG-determined, angle. When we plotted the mean across all flies of these EPG-phase-aligned bumps (Figures 2D and S2A), we observed that the PFNa bumps in both the left and right bridge had a sinusoidal shape, consistent with these signals encoding 2D vectors (Figure S2H). Additionally, we found that, as in the example fly, the PFNa bumps could exist both in phase and antiphase with the EPG heading signal, contingent on the egocentric airflow direction (Figures 2D, 2E, and S2A). With air puffs from the front, both the left and the right PFNa bumps had their peaks aligned with the EPG peaks. With air from the side, the PFNa sinusoid contralateral to the stimulated side of the body had its phase offset by ~180° relative to the EPG bump (Figures 2D and 2E). With air from behind, both PFNa sinusoids expressed an antiphase relationship to the EPG bump (Figures 2D and 2E). Thus, both PFNa sinusoids in the bridge invert, or equivalently shift their phase by ~180°, when air puffs arrive from the back and one or the other sinusoid inverts when air comes from the side.

PFNa cells project axons from the protocerebral bridge to the fan-shaped body (Figure 1C) with an anatomical offset that shifts the peak of each PFNa sinusoid––and thus the angle of the encoded vector––by ~45° clockwise for right-bridge PFNa cells and ~45° counterclockwise for left-bridge PFNa cells^4^ (Figure 1D, solid lines). When a PFNa sinusoid is phase inverted relative to the EPG bump in the bridge, this adds an additional 180° to the encoded phasor’s angle, rotating these vectors from ±45° to ±135° orientations (Figure 1D, dotted lines). Thus, the anatomy and physiology together point to a system in which two PFNa phasors signal the projection of the airflow vector onto two orthogonal axes, and that when this projection requires one or both PFNa vectors to point in the negative direction along their respective axis they can do so. In this way, a two-vector system can signal air puffs both from the front and from the rear.

Whereas the above logic explains the direction of the PFNa vectors, it does not reveal information about their length. PFN vector length is reflected in the amplitude of the PFN phasors in the bridge and also in the PFN calcium signals in the noduli^1^, a set of paired structures just ventral to the fan-shaped body (Figure 1A). The entire set of left-bridge PFNa cells innervate the right nodulus, and vice versa (Figure 1C), with the intensity of left and right noduli tracking the amplitude of the right-bridge and left-bridge PFNa vectors, respectively. Thus, if our vector inversion model is correct then as the air direction rotates 360° around the fly, one should observe two angles, offset by 180°, at which the nodulus activity of a PFNa population is high –– once when the air angle aligns with the positive direction of each vector’s projection axis and once when the air angle aligns with the negative direction. Remarkably, we observed two peaks in the PFNa calcium signal in the nodulus as a function of the airflow angle around the fly, for both the left- and right-bridge PFNa cells (Figures 2F, S2B, and S2C). In previously studied PFN cells that encode vectors that do not invert, noduli tuning curves express only a single peak^1,5^.

The entire array of left-bridge PFNa cells receives a common input from a single LNOa cell in the right nodulus and vice versa for right-bridge PFNa cells^4^. These two LNOa cells are the conduits for airflow information to PFNa cells^4,12^. Intriguingly, when we measured the calcium response of LNOa cells, we observed tuning curves to the direction of airflow with only a single peak (Figures S2D and S2E). How is it that PFNa cells show two calcium response peaks in the nodulus as a function of the airflow direction when their inputs should cause them to maximally depolarize at only one preferred angle? Moreover, what is the mechanism that allows the PFNa sinusoids in the bridge to become phase inverted relative to the EPG signal when airflow arrives from certain directions and not others? We reasoned that electrophysiological measurements from PFNa cells might provide insight into these questions.

### PFNa neurons exhibit two types of spikes poised to underlie signaling of phase-aligned and phase-inverted vectors

**Figure 3.**
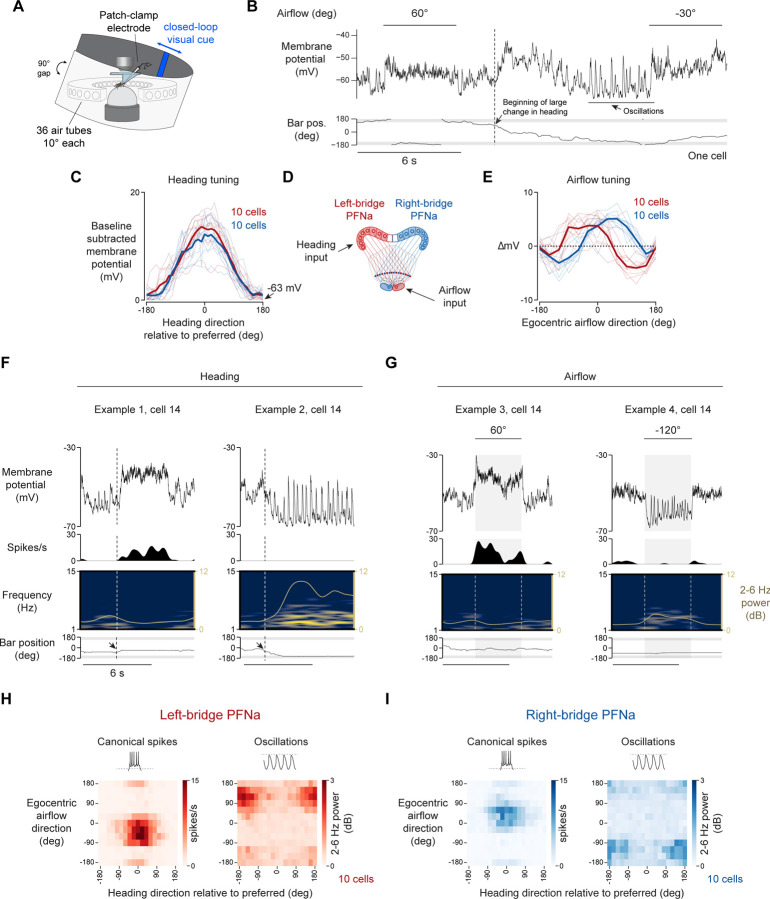
PFNa neurons fire canonical spikes when depolarized and express oscillations when hyperpolarized, with the oscillations signaling airflow stimuli from the egocentric rear. **(A)** Experimental setup. **(B)** Example trace showing the membrane potential (*Vm*) of a right-bridge PFNa neuron. Air puff moments indicated. The angular position of the closed-loop bar on the visual display, which tracks the fly’s heading, is shown underneath. We highlight a moment of a large heading change (dotted line) and a moment showing oscillations in the cell’s *Vm*. **(C)** Tuning of PFNa *Vm* to the fly’s heading, as estimated by the angular position of the closed-loop bar on the visual display. All tuning curves have been phase-aligned to have their peak at 0° (Methods). Left-bridge PFNa neurons: red. Right-bridge PFNa neurons: blue. Thin lines: single fly averages. Thick lines: population averages. We indicate the mean *Vm* at baseline on the right (–63 mV). **(D)** Schematic of PFNa neurons, showing our color conventions. **(E)** Airflow direction tuning curves of PFNa neurons. These tuning curves have not been phase-nulled. Thin lines: single fly averages. Thick lines: population averages. **(F)** Example traces from the same PFNa neuron shown in in panel B. We isolated two moments in time where the fly turned on the ball and thus changed heading (dotted lines). In one moment, this heading change caused the cell to depolarize and fire sodium spikes (left). In the other moment, the fly’s turn caused the cell to hyperpolarize and express large *Vm* oscillations (right). **(G)** Example traces from the same PFNa neuron shown in in panels B and F. We isolated two moments where the fly experienced airflow stimuli (gray box) that strongly altered the PFNa cell’s *Vm*. With one airflow stimulus, the cell depolarized and fired sodium spikes (left). With the other airflow stimulus, the cell hyperpolarized and expressed large *Vm* oscillations. **(H)** Conjunctive tuning of left-bridge PFNa-cell activity to the direction of airflow and heading. Each two-dimensional heatmap shows the population averaged tuning of 10 cells to the two variables. Heading tuning data from individual cells were rotated to make 0° the preferred heading angle, prior to averaging across flies. The left panel shows the tuning of the canonical, sodium-spike signal across the cell population. The right panel shows the tuning of the noncanonical, oscillatory signal across the cell population, using the power in the 2–6 Hz band of the *Vm* as a proxy for oscillation strength (Methods). **(I)** Same as panel E, but for the right-bridge PFNa neurons.

We performed whole-cell patch-clamp recordings from PFNa neurons in head-fixed flies navigating with a closed-loop bar while they also received air puffs from various directions (Figures 3A and 3B). Consistent with past reports^12^, we observed strong responses from PFNa cells to air puffs, both in their membrane potential (*Vm*) and in their spike rate (Figure 3B, air-puff periods; note that sodium spikes in PFNa cells have a very small amplitude when recorded at the soma and are hardly visible at the scale used in Figure 3B). The *Vm* of PFNa cells was not only sensitive to air puffs, but also to changes in the fly’s heading, as signaled by changes in the angular position of the closed-loop bar (Figure 3B, vertical dotted line). Consistent with the example trace, in a population of PFNa cells we observed strong tuning at the level of the mean *Vm* to both the fly’s heading as well as the direction of airflow (Figures 3C, 3E, S3A, S3B, S3D, and S3E). The *Vm* tuning curves for both variables conformed well to sinusoidal functions (Figure S2I), as would be expected from neurons that participate in encoding vectors via a phasor representation.

Conjunctive tuning to allocentric heading and the egocentric airflow direction is what one would expect from a set of PFN neurons that aim to transform the airflow direction experienced by a fly from egocentric to allocentric coordinates. However, these results were initially confusing because they revealed PFNa cells to have *Vm* tuning curves to airflow with only a single peak, at approximately ±45° (Figure 3E), whereas the same cells showed double-peaked tuning curves to the same stimuli when measuring calcium in the noduli (Figure 2F). If a left-bridge PFNa cell’s *Vm* is most hyperpolarized when an air puff arrives from the fly’s back right (i.e. ~100–150°, Figure 3E), how is it that we observe elevated calcium for air puffs at these angles in the population to which this cell belongs?

In the course of performing these experiments, we noticed that when PFNa neurons were strongly hyperpolarized, their membrane potential showed large periodic oscillations (Figure 3B). The oscillations had a periodicity of ~2–6 Hz (Figures S4A and S4B, right panels), which is a frequency range previously reported to be enhanced in the *Vm* of PFNa cells^12^. Using power in the 2–6 Hz band as a quantitative measure of the oscillation strength, we noted robust *Vm* oscillations whenever the membrane potential of PFNa cells was sufficiently hyperpolarized, independently of whether the hyperpolarization was caused by the fly changing its heading toward a non-preferred direction (Figure 3F, right) or by an air puff from a non-preferred angle (Figure 3G, right). Similarly, PFNa cells expressed canonical sodium spikes when their *Vm* was sufficiently depolarized, independently of whether this depolarization was due to the fly’s change in heading or an air puff (Figures 3F and 3G, left).

To evaluate how PFNa neurons respond to combinations of heading and airflow direction, we plotted two-dimensional tuning curves (i.e. heatmaps) of both PFNa canonical spikes and oscillation strength as a function of these two variables (Figures 3H and 3I). We observed the strongest oscillations in response to stimuli that induce the largest membrane hyperpolarization, that is, anti-preferred heading angles and ±120° airflow angles (Figures 3H and 3I, right panels). The airflow directions that elicited *Vm* oscillations matched those that yielded phase inversions of the PFNa sinusoids in the protocerebral bridge in relation to the EPG phase (Figure 2E, 3H and 3I). Conversely, the airflow directions that elicited the strongest canonical spikes in PFNa cells were those that yielded phase alignments between PFNa and EPG bumps in the bridge (Figures 2E, 3H and 3I). A switch in signaling from depolarization-driven canonical spikes to hyperpolarization-driven oscillations is thus poised to underlie vector inversions in the PFNa system. These electrophysiological findings also explain how the LNOa inputs to PFNa cells, which express only a single calcium peak across airflow directions, can drive a two-peaked calcium response in PFNa cells. One calcium peak was likely due to PFNa cells being depolarized and firing canonical sodium spikes. The other peak was likely due to PFNa cells being sufficiently hyperpolarized to express oscillations, which could also lead to elevated calcium.

### Hyperpolarization-elicited oscillations in PFNa cells are mediated by Ca-α1T calcium channels

To test whether the second calcium peak in the PFNa noduli tuning curves stemmed from oscillatory activity, we wished to better understand the mechanism of oscillatory *Vm* dynamics in PFNa cells. We reasoned that if artificially hyperpolarizing a single PFNa cell could reliably trigger oscillations, this would suggest a role for intrinsic membrane conductances in the phenomenon. Consistent with this notion, we were able to routinely induce oscillations in PFNa neurons by injecting hyperpolarizing current into PFNa cells (Figures S4C, S4D, and S4G).

**Figure 4.**
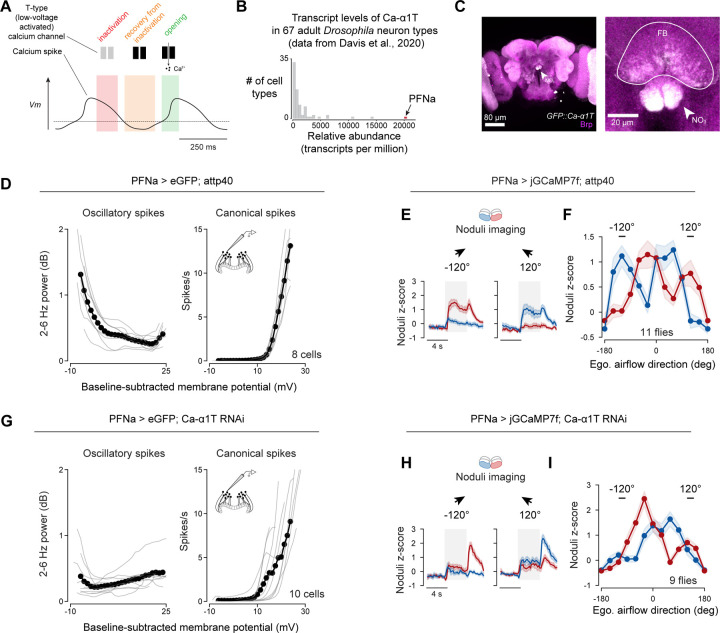
The T-type calcium channel Ca-a1T mediates oscillations in PFNa neurons and thus the ability of these cells to signal rear airflow stimuli. **(A)** Schematic of how T-type calcium channels might contribute to noncanonical calcium spikes, based on the physiology of cat thalamocortical neurons^7^. T-type channels inactivate at depolarized *Vm* and recover from inactivation upon membrane hyperpolarization. After sufficient time at a hyperpolarized *Vm*, a large enough pool of channels is relieved from inactivation, enabling the cell to fire a calcium spike (two shown). In some systems, at the top of each T-type spike, the cell will fire a barrage of sodium action potentials. **(B)** Relative abundance of the RNA transcripts encoding the Ca-α1T channel in a published dataset of 67 cell types in the adult *Drosophila* brain^14^. The relative abundance of the *Ca-α1T* transcript in PFNa neurons is indicated (arrow). **(C)** Immunohistochemically-amplified GFP signal in the brain of an example fly expressing a GFP-tagged Ca-α1T channel (i.e., a GFP::Ca-α1T fusion protein)^15^. The same staining pattern was observed in two additional brains (data not shown). A neuropil stain (bruchpilot) is shown in magenta and GFP fluorescence is shown in white. **(D)** 2–6 Hz power (left panel) and canonical spike rate (right panel) as a function of membrane potential in PFNa neurons recorded in flies from the empty-RNAi control genotype (the genetic background used to create the TRiP RNAi libraries^16^, but where eGFP was expressed in PFNa neurons). Responses of right- and left-bridge PFNa neurons were pooled. Thin lines: single fly averages. Thick lines: population averages. **(E)** Temporal profile of jGCaMP7f responses in the noduli from PFNa neurons. Shown is the population averaged signal from flies expressing GCaMP7f and an empty-RNAi construct in PFNa cells. The time course of the calcium signal at two airflow angles (−120° and 120°) is shown. The thick lines represent the population-mean z-score value and the error regions indicate s.e.m. **(F)** Population-averaged noduli tuning curves of PFNa jGCaMP7f responses. We averaged the z-scored calcium signal value between 2 and 4 seconds after the onset of airflow. Thick lines represent the population-averaged z-score calcium signal. Error bars: s.e.m. **(G)** Same as in panel F, but in PFNa cells carrying the construct TRiP.HMS01948^16^, which allows for expression (under UAS control) of a double-stranded RNA that targets Ca-α1T transcripts for degradation (Ca-α1T RNAi). **(H)** Same as in panel E, but in PFNa cells expressing jGCaMP7f and TRiP.HMS01948 (Ca-α1T RNAi). **(I)** Same as in panel F, but in PFNa cells expressing jGCaMP7f and TRiP.HMS01948 (Ca-α1T RNAi).

The oscillations in the *Vm* of PFNa neurons reminded us of the burst firing mode of mammalian thalamocortical neurons, where low-voltage activated, T-type calcium conductances are key to the production of rhythmic spiking^6,7^ (Figure 4A). Specifically, hyperpolarization of thalamic neurons relieves T-type channels from inactivation, enabling them to produce regenerative calcium spikes^6,7^. The *Drosophila* genome encodes a single T-type calcium channel, Ca-α1T, and a recently published data set revealed that PFNa cells express the *Ca-α1T* transcript at a 35-fold higher level than other cell types analyzed in that study^14^ (Figure 4B). In addition, we observed strong immunohistochemical signal from a GFP-tagged knock-in allele of Ca-a1T^15^, *GFP::Ca-α1T*, in the third layer of the noduli and the ventral layers of the fan-shaped body, which are regions innervated by PFNa neurites (Figure 4C). These observations suggested that a Ca-α1T-mediated conductance may endow PFNa neurons with the ability to oscillate when hyperpolarized.

We knocked down the transcript levels of Ca-α1T in PFNa neurons using RNAi^16^ and recorded PFNa *Vm* while presenting open-loop air puffs to flies navigating a virtual environment. Unlike in control flies, PFNa neurons with *Ca-α1T*-knockdown rarely expressed any *Vm* oscillations to airflow stimuli arriving from behind (Figures 4D, 4G, S3G and S3H) or when we hyperpolarized the cells with current injection (Figures S4E, S4F, S4H, and S4I). PFNa neurons with *Ca-α1T*-knockdown also showed diminished secondary calcium peaks, specifically to airflow stimuli arriving from behind, in their calcium tuning curves measured in the noduli (Figures 4E, 4F, 4H, and 4I). Together, these results demonstrate a critical role for T-type channels in generating the *Vm* oscillations of PFNa neurons, and thus we will refer to these oscillations as calcium spikes hereafter. These results also implicate the *Vm* oscillations in generating the calcium signals associated with air puffs from behind, which underlie the PFNa system’s ability to invert its encoded vectors.

### A qualitative model for how PFNa neurons can invert their encoded vector

**Figure 5.**
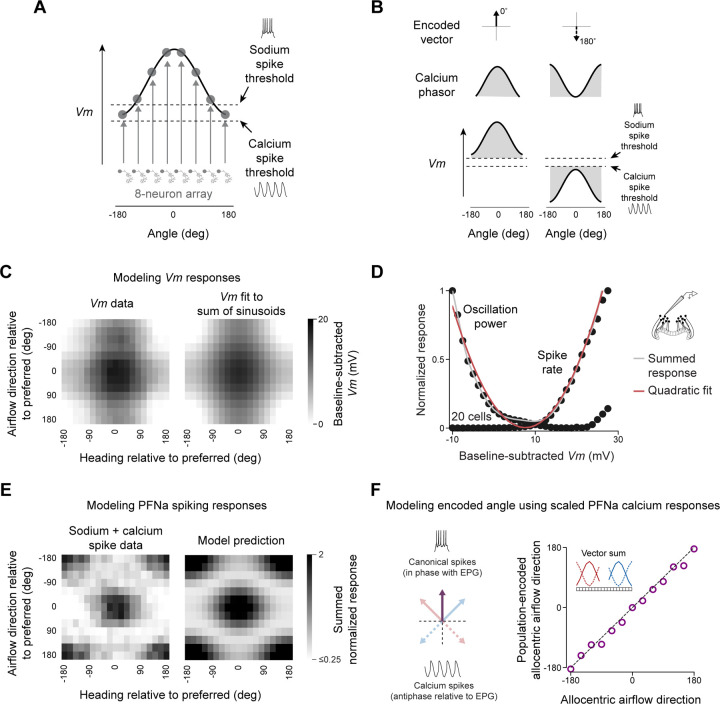
A qualitative and quantitative model for vector computation with invertible vectors in PFNa neurons. **(A)** Schematic of the population activity pattern of a set of eight central complex neurons, arrayed across a structure (e.g., one side of the protocerebral bridge or the fan-shaped body). A sinusoidally modulated pattern of activity across the population is reflected in the *Vm*. We assume that the population has two spike thresholds. One, canonical, sodium spike threshold and one, non-canonical, calcium-spike threshold. **(B)** If an external input uniformly depolarizes the population, the sinusoidal *Vm* signal of the population uniformly rises (left column, bottom). The further above the sodium-spike threshold that a neuron’s *Vm* is, the stronger it will fire sodium spikes and the more calcium will enter the cell. Thus, with uniform depolarization of the population, one expects a sinusoidal, or nearly sinusoidal, calcium signal across the population with a peak at 0° (left column, middle). If we assume that the sinusoidally modulated calcium signal encodes a vector, then the vector would have an angle of 0° (left column, top). Conversely, if an external input uniformly hyperpolarizes the population, then the sinusoidal *Vm* signal across the population will drop (right column, bottom). The further below the calcium-spike threshold a neuron’s *Vm* is, the stronger its T-type spike signal will be and the more calcium will enter the cell. Thus, with uniform hyperpolarization of the population, one expects a sinusoidal, or nearly sinusoidal, calcium signal across the population with a peak at 180° (right column, middle). If we assume that the sinusoidally-modulated calcium signal encodes a vector, then the vector would have an angle of 180°, which is phase-inverted relative to the original case (left column, top). **(C)** Comparison of the airflow-nulled and heading-nulled normalized *Vm* responses of the PFNa neurons (left panel) and the fit to a sum of two sinusoids representing heading and airflow, respectively (right panel, see Methods). The 2-D histograms show data across 18 bins of heading directions (x axis) and the 12 tested airflow directions (y axis). To align data for all cells, we nulled the heading by shifting the data to center each cell’s preferred direction at 0°, and we nulled the airflow responses by first mirroring the data for the left-bridge cells, and then applying a −45° shift to the pooled data for both hemispheres. **(D)** Sodium and calcium spiking responses of the PFNa neurons as a function of membrane potential, mean across 20 cells. Each curve has been normalized to a maximum value of 1 and a minimum value of 0 to highlight the relationship between them despite the difference of their individual units (spikes/s and 2–6 Hz power, respectively). The responses were plotted as a function of the normalized membrane potential, which is simply the membrane potential minus the mV value for the minimum value of the heading tuning curve (Methods). The two normalized curves for spiking rate and 2–6 Hz power were summed (gray line) and a quadratic fit was performed on these values (red line). **(E)** Comparison of the airflow and heading-nulled spiking and oscillation responses of the PFNa neurons (left panel) and the prediction of the quadratic model (right panel). The data panel on the left shows normalized and pooled values for the data in panels 3H and 3I, pooled across brain hemispheres and nulled both by preferred airflow and by preferred heading direction. **(F)** Predicted output direction of the PFNa neurons using the measured, summed calcium responses in Figure S2A (open purple circles). Because the phase-aligned and phase-inverted PFNa signals have different calcium sources and thus different amplitudes in terms of their jGCaMP7f responses, we scaled the phase-inverted calcium responses by a uniform factor of 0.2 to generate this graph.

The findings up to this point allow us to propose a conceptual model for how a population of PFNa neurons can encode a vector whose direction is invertible. Each population of PFNa cells expresses a sinusoidally shaped *Vm* signal across a set of anatomical columns (e.g., across eight glomeruli on one side of the protocerebral bridge or across the columns of the fan-shaped body) (Figure 5A). Two (possibly overlapping) thresholds exist in the system, a sodium-spike threshold in the depolarizing direction and a calcium-spike threshold in the hyperpolarizing direction (Figure 5A). Calcium rises when the *Vm* of a PFNa cell deviates from these thresholds in either direction. Calcium influx occurs with sodium spikes because the membrane is depolarized enough to presumably activate high-voltage activated (HVA) calcium channels. With calcium spikes, calcium influx occurs via the T-type channels directly, or, potentially also indirectly via the activation of HVA calcium channels with sodium spikes that sometimes ride on top of the calcium spikes, a point we return to later. In either case, a calcium bump that encodes a vector is induced across the neuronal population (Figure 5B, middle and top row).

LNOa input to the PFNa population in the nodulus can uniformly depolarize (Figure 5B, left) or hyperpolarize (Figure 5B, right) the PFNa population as a function of the air puff direction. When the PFNa population is uniformly depolarized, the system expresses a sodium-spike induced calcium bump that is aligned with the EPG bump (set to be 0° in Figure 5B, left). When the PFNa population is uniformly hyperpolarized, cells 180° away from the EPG peak are maximally hyperpolarized and the system will generate a T-type spike induced calcium bump whose peak is 180° offset from the EPG bump, thus implementing vector inversion (Figure 5B, right). In this way, an LNOa input signal with a single tuning peak can induce two peaks in the calcium tuning curves of PFNa cells––one peak due to sodium spikes and the other due to calcium spikes––while also inverting the vector encoded by the PFNa population across the two spike modes.

### A quadratic model for computing the allocentric direction of airflow

Motivated by the above concept for how vector sign inversion could work, we aimed to develop a formalism for the computation implemented in the PFNa network. The totality of our experimental results led us to a mathematically concise model for how the egocentric-to-allocentric transformation of airflow direction is accomplished.

The model’s overarching framework is as follows. The PFNa bumps or phasors in the protocerebral bridge are either in phase or ~180° out of phase with the EPG heading angle, thus instantiating invertible vectors. PFNa cells send axons from the bridge to the fan-shaped body with an offset that rotates the left- and right-bridge phasors by ±45°, giving rise to a pair of orthogonal vectors in the fan-shaped body, which form a basis for the allocentric direction of airflow (Figure 1D). The allocentric airflow direction can be estimated by summing the two vectors. Air puffs from the front lengthen the ±45° vectors. Air puffs from the rear invert the vectors such that they shift to pointing at ±135° (Figure 1D), allowing the system to represent directions that have negative projections along the ±45° axes. We present a detailed analysis of these vector angles in the Supplementary Text and Figure S5.

To formalize our model quantitatively, we first focused on our electrophysiological data. The *Vm* of PFNa neurons appeared to be sinusoidally modulated both by the egocentric direction of airflow and by the allocentric heading angle (Figures 3C and 3E). We therefore fit the *Vm* responses of PFNa neurons (Figure 5C, left), over the full range of heading and airflow directions, to a sum of two sinusoids, with one sinusoid representing the airflow response and the other representing the heading response (Figure 5C, right). The resultant, three-parameter fits explain 91% and 94% percent of the variance of the 216 data points in the *Vm* two-dimensional tuning-curve from the left- and right-bridge PFNa populations, respectively (Methods). The quality of these fits support the notion that the heading and airflow inputs combine additively.

To fit the spiking responses of the PFNa cells (Figures 3H and 3I), we made use of the fact that both the sodium-spike rate and the calcium-oscillation strength showed a quadratic dependence on *Vm* (Figure 5D). Using the sum-of-sinusoids model for *Vm*, followed by a squaring operation, we fit both the sodium-spike rate and the calcium oscillation strength across all airflow and heading angles (Figure 5E). Specifically, we modeled the sodium-spike rate response for egocentric airflow angle *W* and relative heading angle *H* as ${\left({\left[b_{H}\;\text{cos}\left(H\right)+b_{W}\;\text{cos}\left(W\pm 45{}^{\circ}\right)\right]}_{+}\right)}^{2}$, where *b*_*H*_ and *b*_*W*_ are fitted constants, []_+_ indicates rectification, and ± refers to left/right PFNa neurons. We modeled the oscillation strength similarly except with reverse rectification for negative values. The model accounts for 87% of the variance for spike rate and 73% for oscillation strength, averaged across left and right PFNa neurons. The model fit the patch-clamp data extremely well for 11 of the 12 air-puff angles tested and thus independently of whether cells were emitting sodium or calcium spikes (Figure 5E). One exception was that air puffs from directly behind the fly yielded a low rate of sodium spikes from PFNa neurons, which our model did not predict (Figures 3H and 3I, left panels). This anomalous measurement is unlikely to reflect a technical artifact of rear airflow not arriving to the fly (Figures S2F and S2G) and thus future work will be needed to explain the observation.

It is not unusual to model firing rates as a quadratic function of *Vm* above some firing threshold but, in the case of PFNa neurons, the existence of calcium spikes introduces the possibility of a full, unrectified squaring operation in terms of the effective output of PFNa neurons (Methods). If we assume that this is the case, the spiking and oscillatory outputs can be combined, yielding a response proportional to the single expression ${\left(b_{H}\;\text{cos}\left(H\right)+b_{W}\text{ cos}\left(W\pm 45{}^{\circ}\right)\right)}^{2}$, similar to what is written above, but without the rectification. If we shift the term cos(*H*) by ±45° to model the left and right PFNa responses in the fan-shaped body, we can write the combined output of a matched pair of PFNa neurons to a downstream target as ${\left({\left[b_{H}\;\text{cos}\left(H-45{}^{\circ}\right)+b_{W}\;\text{cos}\left(W+45{}^{\circ}\right)\right]}_{+}\right)}^{2}+{\left({\left[b_{H}\;\text{cos}\left(H+45{}^{\circ}\right)+b_{W}\;\text{cos}\left(W-45{}^{\circ}\right)\right]}_{+}\right)}^{2}$. Using trigonometric identities, this expression is equal to $b_{H}{}^{2}\;+b_{W}{}^{2}\;+2b_{H}b_{W}\text{cos}\left(W+H\right)$ (Methods). The last term in this expression describes the tuning of the downstream output as being a function of *W* + *H*, the airflow angle in allocentric coordinates, and the full collection of these outputs across the columns of the fan-shaped body is a phasor that represents the allocentric airflow direction (Methods).

Finally, we asked whether we could sum the experimentally measured responses of PFNa neurons, rather than use the above equations, to obtain the allocentric airflow direction. To do this, we used PFNa calcium signals recorded in the protocerebral bridge. Because such signals include calcium due to ordinary spikes as well as calcium spikes, which can have different physiological consequences, we needed to introduce a factor that converts the calcium-spike signal into an equivalent sodium spike signal. Using a factor of 0.2 (Methods), we computed the allocentric airflow direction by summing the left- and right-bridge PFNa phasors appropriately shifted and weighted by this factor and then we determined the phase (or equivalently maximum) of the resulting signal. This algorithm generated an output signal that accurately tracked the actual airflow direction (Figure 5F).

### FC3 cells read out the EPG-phase-aligned vectors during air puffs

**Figure 6.**
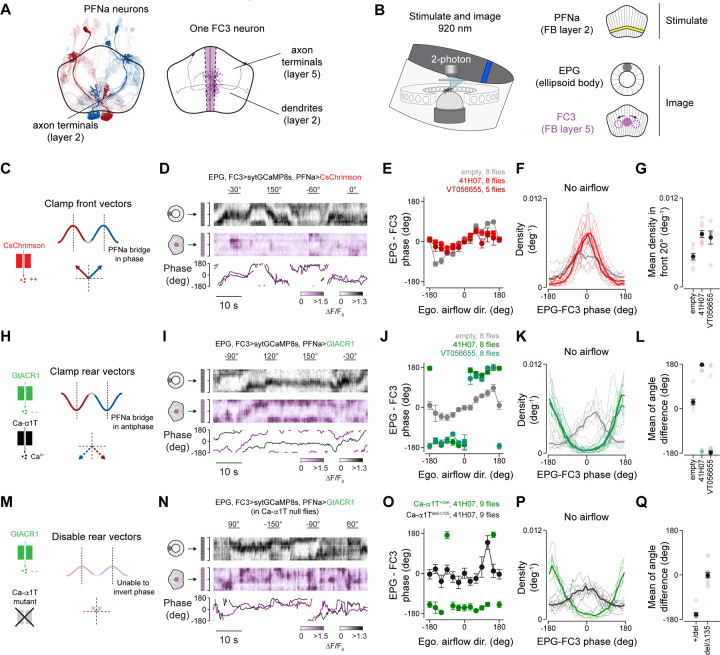
FC3 neurons can functionally sum two sodium-spike or two calcium-spike encoded vectors in the PFNa populations. **(A)** Anatomy of the PFNa and FC3 neurons. PFNa neurons shown at left synapse onto the FC3 neurons. A single FC3 neuron is shown on the right. **(B)** Schematic of the experimental setup. For all the experiments in this figure, we imaged the EPG neurons in the ellipsoid body and the FC3 neurons in the fan-shaped body, while optogenetically stimulating PFNa neurons at their axonal terminals in layer 2 of the fan-shaped body. The bump in the FC3 neurons is aligned across layers 2 (dendrites) and 5 (axonal terminals) of the fan-shaped body (data not shown); in these experiments, we report the FC3 axonal signals in layer 5 because they were easier to segment accurately. **(C)** Schematic of the expected effect of depolarizing PFNa neurons with CsChrimson on the sinusoidal signals that PFNa populations express and the vectors that these population signals encode. The sinusoids shown schematize the population activity pattern of PFNa neurons in the protocerebral bridge, with the dotted lines represent the position of the EPG bumps. **(D)** Example EPG and FC3 bumps in the context of optogenetic depolarization of PFNa neurons (41H07-Gal4 > UAS-CsChrimson-tdTomato) (top). Extracted bump phases are shown at bottom. **(E)** FC3 – EPG phase in the context of airflow stimuli during optogenetic depolarization of PFNa neurons. The values shown here correspond to the average phase difference over the last two seconds of the airflow stimulus. Error bars represent s.e.m. The three Gal4 lines used to drive effector expression are written above the panel. **(F)** Probability distributions of the EPG-FC3 phase difference in the context of optogenetic depolarization of PFNa neurons. The data shown here are taken from time periods with no airflow. Thin lines: single fly averages. Thick lines: population averages. The three Gal4 lines used to drive effector expression are written above the panel. **(G)** Mean density in the frontal 20° of the EPG-FC3 phase difference (average of points ±10° from 0° in the x axis in panel F. Error bars show the s.e.m. The three Gal4 lines used to drive effector expression are written above the panel. **(H)** Same as in panel C, but for optogenetic hyperpolarization of the PFNa neurons via GtACR1. **(I, J, K)** Same as in panels D, E, and F, but for optogenetic hyperpolarization of PFNa neurons via GtACR1. **(L)** Circular difference of the average density of EPG-FC3 phase difference curves in shown in panel K. Error bars: s.e.m. **(M)** Same as in panel H, but in the context of flies harboring a null mutation of Ca-α1T (Ca-α1T^del/Δ135^). **(N, O, P, Q)** Same as in panels I, J, K, L) but in the context of flies harboring a null mutation of Ca-α1T (Ca-α1T^del/Δ135^).

Our modeling work argues that the PFNa vectors can, in principle, be read out to track the allocentric direction of air puffs. We wished to understand if neurons that are monosynaptically downstream of PFNa cells actually perform such a read-out operation. PFNa neurons synapse onto ten classes of columnar cells in the fan-shaped body^4^ (Figure S6A). We chose to focus on FC3 neurons (Figure 6A) because their characteristic anatomy aided us in genetically targeting them with high specificity, and also because the majority of the heading inputs to FC3 cells are mono- or disynaptically received from PFNa cells^4^, rather than from other PFN types (Figure S6A).

We imaged calcium in FC3 and EPG neurons as head-fixed flies navigated a virtual environment and received air puff stimuli from 12 directions. Akin to EPG cells in the ellipsoid body, FC3 cells express a single bump of calcium activity whose left/right position, or phase, in the fan-shaped body changes over time (Figure S6B). Prior to the first air pulse and during inter-air-puff intervals, the phase of the FC3 bump was not consistently aligned to the EPG phase (Figure S6B, purple and black curves). During air-puff stimuli, the FC3 bump amplitude increased and its phase systematically realigned in reference to the EPG phase (Figure S6C). Specifically, in an analysis of 7 flies we found that the FC3 phase deviated from the EPG phase in linear proportion to the egocentric angle of the air puff for puffs delivered within ±55–60° of the midline. For air puffs delivered more peripherally than ~55° from the midline (i.e., puffs from the sides and rear), the FC3 phase did not systematically deviate beyond 55–60° (Figures S6D and S6E). With air puffs directly from the back, we did not observe a consistent difference between the EPG and FC3 phases (Figures S6D and S6E). Given the anatomy and physiology of the PFNa system described above, these data are consistent with the PFNa front vectors, encoded by sodium spikes, being able to reposition the FC3 bump in the fan-shaped body. However, the inverted, rear-facing vectors, encoded by PFNa calcium spikes, did not appear to influence the FC3 bump in these experiments because the FC3 phase did not consistently deviate more than ±55° from the midline when flies were presented with air puffs from the side and back. (See Figure S5 and Supplementary Text for why the front vectors, alone, could enable deviations of up to ±55° rather than just up to ±45°.)

### FC3 cells read out the EPG-phase-aligned and EPG-phase-inverted PFNa vectors during optogenetic stimulation of PFNa cells

One reason for why the FC3 bump did not accurately signal the allocentric angle of air puffs from the back could be that flies need to be in a specific behavioral state for PFNa calcium spikes to impact downstream circuitry. If so, different tasks or stimuli might ultimately reveal cases in which the inverted vectors induce downstream effects. Because we do not yet know the nature of such putative tasks and/or stimuli, we reasoned that optogenetic perturbations of the PFNa population might serve as another fruitful approach for testing whether FC3 neurons can sum both the phase-aligned and phase-inverted PFNa signals.

According to our model, uniform depolarization of both PFNa populations should extend the two EPG-phase aligned vectors. If the activity of FC3 neurons reflects the sum of these two vectors, the phase of the FC3 bump should align with the EPG phase during such PFNa depolarization. To test this hypothesis, we optogenetically depolarized PFNa cells while simultaneously imaging the FC3 and EPG calcium bumps (Figures 6A-6C). To depolarize PFNa cells, we expressed the cation-permeable channelrhodopsin CsChrimson^17^ in them and to image the FC3 and EPG bumps, we expressed jGCaMP8s^18^ in these cells (Figures 6B and 6C). With the same 920 nm illumination light, we could simultaneously stimulate PFNa axons in the fan-shaped body, and image calcium in FC3 neurons in the fan-shaped body and EPG neurons in the ellipsoid body (Figure 6B). We found that with optogenetic depolarization of PFNa cells, the FC3 and EPG phases became more aligned (Figures 6D-6G), consistent with our model. This effect was clear both during airflow (Figure 6E)––for which we observed muted deviations of the FC3 phase from the EPG phase for air puffs from the sides and rear––and during moments when no airflow was presented (Figures 6F and 6G).

We also performed the converse experiment, optogenetically hyperpolarizing PFNa cells while simultaneously imaging the FC3 and EPG calcium bumps (Figures 6H-6L). Uniform hyperpolarization of both PFNa populations should induce calcium spikes in PFNa cells and thus extend the two EPG phase-inverted vectors (Figure 6H). If the inverted vectors can impact downstream physiology, the phase of the FC3 bump should show a 180° offset relative to the EPG bump during such a perturbation. To hyperpolarize PFNa cells, we expressed the chloride-selective channelrhodopsin GtACR1^19^ in them. Otherwise, the experiment we performed was identical to the CsChrimson experiment just described. We found that with PFNa cells hyperpolarized, the FC3 bump was consistently antiphase to the EPG bump (Figures 6I-6L), supporting our model and providing evidence for the ability of the calcium-spike-associated vectors to impact downstream physiology. This effect was clear both during air puffs (Figure 6J) and during moments when no air puffs were presented (Figures 6K and 6L).

Eliminating the ability of neurons to express T-type calcium spikes should eliminate phase-inversion of the FC3 bump relative to the EPG bump with PFNa hyperpolarization. We repeated the experiment in Figure 6H in Ca-α1T-null flies (Ca-α1T^del/Δ135^), which are expected to lack functional T-type calcium channels^15,20^ (Figure 6M). Consistent with our model, the FC3 bump no longer appeared phase inverted relative to the EPG bump in these experiments (Figures 6N-6Q). These results support the hypothesis that PFNa calcium spikes can induce postsynaptic FC3 activity, with the phase of the FC3 bump reflecting a vector sum of the two phase-inverted vectors expressed in the PFNa populations. We verified that optogenetic hyperpolarization of PFNa neurons induces T-type calcium spikes in non-mutant flies (Figure S4K). In the Discussion, we consider why these spikes might have led to fast transmitter release in the optogenetic experiments but not in in the air-puff experiments. Taken together, our optogenetic experiments demonstrate that both EPG phase-aligned and EPG phase-inverted vectors in PFNa cells can induce vector-addition-like signaling in downstream cells.

## Discussion

### What is the function of PFNa cells?

PFNa cells receive inputs related to the fly’s allocentric heading angle in the protocerebral bridge and inputs related to the egocentric direction of airflow in the noduli (Figure 1C). Using these inputs, two PFNa populations––one originating in the left bridge and the other in the right bridge––generate two calcium signals that are sinusoidally modulated across space, which function as invertible vectors (Figures 2 and 3). By summing the two invertible vectors signaled by the two PFNa populations, the allocentric direction of airflow (or, potentially, other directional stimuli that activate PFNa cells) can be calculated (Figure 5F). In situations where flies are standing still, or walking slowly, the direction of airflow sensed by the body directly reflects the direction of wind in the external world. The direction of wind is of broad importance to navigating insects^21–29^, and thus this circuit has the potential to generate a signal^30^ that can guide many homing and food-orienting behaviors that rely on wind assessments. Indeed, impairing PFNa physiology has been shown to impact airflow related orienting in tethered, flying flies^12^.

The airflow tuning of PFNa cells likely originates from indirect inputs that these cells receive from the antennae^4,31,32^. Airflow tuning makes sense because each antenna houses a directional airflow sensor called the Johnston’s organ^33,34^. Beyond airflow, the *Drosophila* antennae also house sensors for temperature^35,36^, sound^37,38^, and the angle of the fly’s body in relation to gravity^39,40^. If PFNa neurons were to receive inputs related to these other variables, they could transform angles associated with those modalities into allocentric coordinates as well. It is also possible that PFNa cells function to transform an “angle of current relevance” into allocentric coordinates and that this angle transcends any specific sensory modality. In this view, our air puff stimuli simply emphasized one direction around the fly as being important in the moment, thus engaging the PFNa network. Regardless of the ultimate function(s) of PFNa cells, our work reveals that these neurons can implement an egocentric-to-allocentric coordinate transformation by instantiating invertible vectors.

### A common allocentric reference frame in the fan-shaped body

The egocentric-to-allocentric coordinate transformation of the air puff direction implemented by PFNa cells is analogous to the coordinate transformation implemented by other PFN neurons in relation to the fly’s traveling direction^1,5^. The fan-shaped body thus seems to house multiple calcium bumps that track navigationally relevant variables in a common allocentric reference frame. The positions of bumps along the left-right axis of the fan-shaped body map to allocentric angles in the outside world (i.e., north, east, south, west, etc.)^1,5,41,42^. Having multiple variables, or calcium bumps, expressed in a common allocentric reference frame is likely to facilitate downstream navigational calculations. By storing a trace of a given bump’s position in the fan-shaped body, for example, a fly could store a memory of an angular direction of relevance, thus allowing the fly to use variables dissociated from its current orientation or sensory experience^43^. There are seven additional PFN cell types in the *Drosophila* central complex that have yet to be studied physiologically^4^. An important next step is to identify the variables that these other PFN neurons place within this common reference frame.

### Synaptic transmission and T-type calcium spikes

Classic synaptic release relies on calcium entry through high-voltage-activated calcium channels, which are found very close to the synaptic-vesicle fusion machinery^44,45^. Because low-voltage activated calcium channels, like T-type channels, do not typically reside immediately adjacent to active zones, calcium entry through these channels is not commonly thought to drive vesicle fusion. The air-puff responses of FC3 neurons, which are monosynaptic recipients of PFNa input, generally support this dichotomous view of calcium signaling at PFNa synapses. That is, the phase of the FC3 bump quantitatively tracked the allocentric direction of air puffs when these arrived to the front of the fly and were thus signaled by presynaptic sodium spikes; however, the phase of the FC3 bump did not properly track the allocentric direction of air puffs when these arrived to the rear of the fly and were thus signaled by presynaptic calcium spikes (Figure S6E). With open-loop air puffs, it seemed that only the sodium-spike-mediated PFNa vectors were able to influence the phase of the downstream FC3 cells.

In contrast to open-loop air puffs, the FC3 phase was consistently offset by ~180° from the EPG phase––thus signaling the rearmost allocentric angle––when we induced calcium spikes via optogenetics in both the left and right bridge PFNa populations (Figure 6K). Notably, we often observed sodium action potentials expressed at the peak of calcium spikes when we injected hyperpolarizing current into PFNa cells with a patch pipette (Figure S4J), but we did not typically observe such sodium spikes with air puff-induced hyperpolarization of PFNa neurons. We thus speculate that we reliably triggered sodium action potentials at the peak of each calcium spike with two-photon optogenetic stimulation of PFNa cells. With rear air puffs, on the other hand, we may not have triggered any sodium spikes on top of the calcium spikes, and the calcium spikes acting alone may not have been able to induce synaptic transmission. One idea is that a specific modulator state was not engaged during our open-loop air puffs, which muted the expression of sodium spikes and thus synaptic transmission. Optogenetic stimulation, on the other hand, may have been sufficiently strong to bypass the need for such a modulatory input. (Because the amount of hyperpolarization that chloride channels can evoke is limited, our optogenetic stimulation is likely to have induce a physiologically relevant state.) Sodium spikes riding on T-type calcium spikes have been observed in other types of *Drosophila* neurons^46^.

Because sodium spikes do not always ride on top of calcium spikes in PFNa neurons, and because calcium spikes have very different timescales and potential downstream effects than sodium spikes, it is important to note that our results to date are agnostic as to whether the central complex can combine one sodium-spike (phase-aligned) phasor (say, in the left bridge) with one calcium-spike (phase-inverted) phasor (say, in the right bridge) to perform a vector sum. Our quantitative model argues that this sort of interaction has the potential to generate an accurate allocentric angle as its output––with appropriate weighing of the two signals––but it is possible that the two modes of signaling do not interact in the real brain. Future work will be needed to resolve this matter.

Beyond eliciting fast neurotransmitter release from clear synaptic vesicles, the wide (~200 ms) calcium spikes elicited by T-type calcium channels are also well suited for promoting peptide release from dense core vesicles^47–49^. The release of neuropeptides into the fan-shaped body might not be immediately apparent at the level of postsynaptic calcium. Rather, neuropeptides may elicit downstream molecular processes that are sinusoidally modulated in their intensity across the left/right extent of the fan-shaped body, representing a vector memory in the system that can alter navigation-related computations at a later timepoint.

### Vector integration and T-type calcium spikes

T-type calcium spikes in PFNa neurons might also serve intracellular signaling roles, rather than, or in addition to, synaptic transmission-related roles. For example, calcium-calmodulin dependent protein kinase II (CaMKII) is particularly sensitive to calcium oscillations at the 2–6 Hz frequency range^50^, which matches the calcium spike rate of PFNa neurons. CaMKII might therefore integrate the information available in calcium spikes over time. Because the calcium spike amplitude (i.e., the power of the 2–6 Hz *Vm* oscillation) and thus calcium influx at 2–6 Hz, varies sinusoidally across the left/right extent of the fan-shaped body, a CaMKII integral of this process––expressed across the array of PFNa axon terminals in the fan-shaped body––could represent a vector that grows in amplitude with each T-type spike across the population. Such an integrated vector could indicate for how long airflow has arrived at the fly’s rear, for example, which might be useful for driving orienting behaviors. Past work had hypothesized that PFN neurons might function as vector integrators^3^ and the calcium spikes described here provide a potential mechanism for this idea to be implemented. Calcium potentials could also mark synapses as being eligible for plasticity^51^ (e.g. via CaMKII phosphorylation^52^), creating a sinusoidally modulated vector-trace signal across the fan-shaped body in this manner as well.

### Neural computation and T-type calcium spikes

Beyond the fly central complex, broad calcium spikes have been observed in the giant motor axons of the jellyfish *Aglantha* and in neurons of the mammalian inferior olive^53^. Delta rhythms in thalamocortical networks, which rely on T-type calcium channels^54,55^, are a famous correlate of sleep^7^. Hippocampal and cortical networks express oscillatory dynamics in the delta and theta range during navigational tasks, and the functions of these oscillations are still being studied^56–58^. Our work shows that T-type calcium channels serve a quantitatively precise vector-computing function in the central complex of *Drosophila*. Similarly explicit, real-time computational functions for calcium spikes likely await discovery in other neurobiological systems as well.

## Methods

### Fly husbandry

Unless indicated otherwise, flies were reared in standard cornmeal-agar-molasses food in a 12h/12h light cycle incubator set to 25 °C. Progenies from crosses were transferred into fresh vials on the day of eclosion and housed in an incubator for 2–7 days before being affixed to a physiology platform for calcium imaging. For electrophysiological experiments, we used 4–7 day old flies.

Flies being crossed for optogenetic experiments were raised in cornmeal-agar-molasses containing vials, wrapped in aluminum foil to minimize light exposure during development. On the day of eclosion, newly hatched flies were transferred into cornmeal-agar-molasses containing vials, supplemented with 0.4 mM all-trans retinal (Sigma Aldrich). These vials were wrapped in aluminum foil for 2–5 days, until flies were affixed to a physiology platform for imaging experiments.

### Fly stocks

Genotypes for each experiment are listed in Table 1. Stock sources are listed in Table 2.

We used existing Gal4 and LexA driver lines^60–64^ to target transgene expression to central-complex neurons. We used the 12E04-LexA driver line to target FC3 neurons, and we used the 27F02-LexA, 60D05-LexA, and 60D05-Gal4 lines to target EPG neurons. In addition to targeting EPG neurons, we found that the 60D05-LexA and 60D05-Gal4 lines also target unidentified cells that innervate layers 2 and 5 of the fan-shaped body, which have airflow responses (data not shown). Thus, whenever we used 60D05-Gal4 or 60D05-LexA to image EPG neurons in conjunction with either PFNa or FC3 neurons, we expressed the red calcium indicator jRGECO1a^65^ to avoid uncertainty as to the cellular origin of fluorescence signals in the fan-shaped body. In optogenetic experiments––where it was not possible for us to work with two different calcium indicators––we used 27F02-LexA to drive syt-jGCaMP8s (based on jGCaMP8s^18^ and described below) expression in EPG cells because this driver line does not target airflow-responsive fan-shaped body cells. For all other imaging experiments, we used jGCaMP7f^66^ instead.

We targeted PFNa neurons using four different driver lines: the split-Gal4 SS02255^67^, 30E10-Gal4, 41H07-Gal4, and VT056655-Gal4. From inspection of the publicly available multi-color Flp-out images by Janelia Research Campus^68^ as well as from our own immunohistochemistry data, we believe that PFNa neurons are the only PFN cells targeted in these four driver lines. We found that using 30E10-Gal4 to drive UAS-GtACR1 expression was lethal at the pupal stage, and thus we used 41H07-Gal4 and VT056655-Gal4 for the experiments in Figure 6. We targeted the LNOa neurons using split-Gal4 SS047432^67^.

We designed the syt-jGCaMP8s construct by linking the *Drosophila* synaptotagmin-1 coding sequences and jGCaMP8s (Addgene Plasmid #162380)^69^ using a GSGSGS linker, with the Syt1 sequence at the C terminus. We then placed this construct into pJFRC19–13xLexAop2 backbone (Addgene Plasmid #26224)^60^, replacing myrGFP with syt-jGCaMP8s. The plasmid was synthesized by GenScript and inserted in the VK00022 landing site (BDSC Stock #9740) using PhiC31-based integration, performed by BestGene.

10xUAS-GFlamp1 was created using an attB-site carrying plasmid gifted to us by Yulong Li’s research group^70^. We inserted the plasmid at the VK00005 integration site (BDSC #9725) with PhiC31-based integration, performed by BestGene.

### Fly mounting

We cold-anesthetized and mounted adult female flies to a custom stage, which allows for head-fixed behavior simultaneous with neural imaging as described previously^73^. In brief, we attached the dorsal tip of the head and the anterior tip of the thorax to a form-fitting hole in the stage using a blue-light-activated glue (Bondic). After being thus attached, the posterior edge of the head capsule can be dissected for physiological measurements from the brain. For calcium imaging experiments that did not require optogenetic activation of neurons, we allowed the flies to recover for ≥ 2 hours under low levels of ambient light after being mounted. For experiments with imaging and optogenetics (Figure 6), we allowed the flies to recover for ≥ 2 hours inside a dark cardboard box. For electrophysiology experiments, we allowed the flies to recover for 2–4 hours inside a dark cardboard box. We used slightly different head pitch angles, depending on the brain structure which we needed to access. The angle between the front vertical drop of the thorax and back of the fly’s head in experiments that involved co-imaging the ellipsoid body and the fan-shaped body was ~60° (as in refs. ^1,74^). This same angle was closer to ~45° for experiments in which we performed imaging of the protocerebral bridge and the noduli, or electrophysiology from PFNa somas.

### Extracellular saline composition and delivery

For both imaging and electrophysiology experiments, we exposed the dorsal surface of the fly’s brain by cutting a rectangular window in the head capsule using a 30-gauge syringe (BD PrecisionGlide). We perfused the brain with an artificial extracellular saline solution^75^ bubbled with carbogen (95% CO_2_ / 5% O_2_). The composition of the saline solution, in mM, was 103 NaCl, 3 KCl, 5 N-Tris(hydroxymethyl) methyl-2-aminoethanesulfonic acid, 10 trehalose, 10 glucose, 2 sucrose, 26 NaHCO_3_, 1 NaH_2_PO_4_, 1.5 CaCl_2_, and 4 MgCl_2_. All chemicals were sourced from Sigma Aldrich. The solution’s osmolarity was measured to be ~280 mOsm, and after carbogen bubbling, the solution’s pH was close to 7.3. The saline was delivered to the brain using a gravity-fed perfusion system. Using a Peltier device (SC-20, Warner Instruments) regulated by a closed-loop temperature controller (CL-100, Warner Instruments), we set the saline’s temperature, measured in the bath, to 22°C for calcium imaging experiments and 25°C for electrophysiology experiments.

### Two-photon calcium imaging

Calcium imaging data were acquired using an Ultima IV two-photon microscope (Bruker) powered with a Chameleon Ultra II Ti:Sapphire tunable laser (Coherent). In experiments where GCaMP fluorescence was imaged alone, the laser wavelength was set to 925 nm. In experiments where we simultaneously imaged GCaMP and jRGECO1a (Figures 2 and S2), the Chameleon laser wavelength was set to 1000 nm and supplemental excitation of the jRGECO1a calcium sensor was provided by a coaxial second laser set to 1070 nm (Fidelity-2, Coherent). The power of the Chameleon laser, as measured at the back aperture of the objective, was 20–50 mW for all experiments. Emitted light was collected through a 40x/0.8 NA objective (LUMPLFLN 40XW, model 1-U2M587, Olympus), split by a 575 nm dichroic mirror (575dcxr, Chroma), and collected by a pair of GaAsP photomultiplier tubes (H7422P-40, Hamamatsu). The green channel, capturing the jGCaMP7f or jGCaMP8s emission signal, was filtered through a 490–560 nm bandpass filter (525/70m-2p, Chroma). In dual imaging experiments, the red channel (containing the jRGECO1a signal) was filtered through two stacked 590–650 nm bandpass filters (620/60m-2p, Chroma). The objective was mounted on a Piezo device (525800–400, Bruker), which allowed for rapid scanning along the z axis. We imaged exclusively in galvo-galvo mode and used the Piezo device to acquire imaging volumes consisting of 3 to 6 z-planes scanned at 128×128 pixel resolution. The volumetric scanning rates ranged from 3 Hz––for experiments with a large region of interest (ROI), such as when we co-imaged the fan-shaped body and ellipsoid body (e.g., Figures 6 and S6)–– to 8 Hz when we employed very small ROIs, like when imaging the noduli (e.g., Figures 2 and S2). The dwell time for each pixel ranged from 3.6 to 4.8 μs. These settings minimized fluorophore bleaching while still providing adequate fluorescence signals.

### Optogenetic stimulation

We used the two-photon laser to perform simultaneous two-photon imaging and GtACR1^19^ or CsChrimson^17^ activation (Figure 6), similar to previous approaches^1,41,76^. In these experiments, the laser power was increased from 20 to 50 mW across the six imaging planes at increasing depth, following an exponential trajectory. The 20-mW plane covered the posterior end of the fan-shaped body, and the 50-mW plane covered the anterior end of the ellipsoid body. Such an exponential power increase had several advantages. First, by having the power in the fan-shaped body be relatively low, this limited photobleaching of jGCaMP8s in the fan-shaped body. Second, the low laser intensity in the fan-shaped body should help to lead to moderate, hopefully physiological, excitation levels of optogenetic reagents in PFNa terminals, rather than overactivation. Third, the high intensity in the ellipsoid body helped to increase the quality of the signal from this deep structure. We imaged volumes at ~3.5 Hz in optogenetic experiments. Approximately three planes were dedicated to imaging the fan-shaped body and approximately three planes were dedicated to imaging the ellipsoid body. Because we were optogenetically activating the terminals of PFN neurons in the fan-shaped body, we thus activated PFN cells at ~3.5 Hz and with a ~50% duty cycle.

### Electrophysiology

We cold-anesthetized flies and affixed them to a custom stage as described above and previously^73^. We opened a small cuticular window over the central complex using a 30-gauge syringe (BD) and removed the underlying fat and tracheal tissue, with fine forceps, to expose the brain. We illuminated the fly using an 850 nm LED (M850F2, Thorlabs) coupled to a 400 μm wide fiber optic cable (M28L01, Thorlabs) that was focused onto the fly with lenses (MAP10100100-A, Thorlabs). We visualized GFP-expressing cell bodies via standard epifluorescence, except that we used a custom GFP emission filter, which passed 510–560 nm and >800 nm (Chroma). This filter allowed us to visualize green and infrared while also cutting out the red fluorescence of Alexa-568, which we often included in our pipette for anatomical fills.

We pressure ejected 0.5% collagenase IV (Worthington) in extracellular saline from a pipette with a 4–6 μm tip positioned over the neural lamella and perineurial sheath, which weakened and ultimately breached these layers. We raised the bath temperature to ~30°C during this desheathing process, which took < 5 min., to activate the collagenase. Following rupture of the perineurial sheath, we lowered the bath temperature to 21°C for ≥ 5 min. and increased the flow rate of the perfusion to ensure that the collagenase was fully washed out. We then performed additional manual clearing of tissue with the micropipette containing extracellular saline until individual somas of interests we exposed. We held the temperature of the bath between 24°C - 28°C for the remainder of the experiment.

We used borosilicate glass capillaries (BF150–86-7.5, Sutter Instruments), pulled on a P-1000 puller (Sutter Instruments) and fire polished with a microforge (MF2-LS2, Narishige) to produce pipettes with 6–12 MΩ resistance and ~1 μm tip openings. Pipettes were filled with intracellular saline whose composition, in mM, was 140 potassium aspartate, 1 KCl, 10 HEPES, 1 EGTA, 0.5 Na_3_GTP, 4 MgATP, 13 biocytin hydrazide, and 0.02 Alexa Fluor 568 hydrazide (A10437, ThermoFisher Scientific). The pH of the intracellular saline solutions was adjusted to 7.3 with KOH and its osmolarity was adjusted to 265 mOsm with water.

We visualized somas for targeting with a patch pipette using a Kinetix sCMOS camera (Teledyne Photometrics) mounted on an upright epi-fluorescence microscope (Slicescope, Scientifica) with a 40×/0.80 NA water immersion objective (LUMPLFLN 40XW, Olympus). We took care to only record from GFP expressing cells. Electrophysiological signals were amplified and low pass-filtered at 10 kHz using a MultiClamp 700B amplifier (Molecular Devices). We streamed all voltage and current signals continuously at 10kHz with a Digidata 1440A input-output board (Molecular Devices). All data presented are *Vm* measurements in current clamp mode. We injected a small amount of current (−1 to −3 pA) during *Vm* recordings, to partially address the depolarizing effect of the seal conductance on small cells, with high input resistance. Out of 41 PFNa cell recordings, across all genotypes, in which we were able to present the entirety of the airflow-direction protocol, we excluded one cell from analysis because the fly did not walk enough to allow us to estimate a heading tuning curve. We excluded two additional cells because their baseline membrane potentials were above our threshold *Vm* for analysis, which was –40 mV before liquid-liquid junction potential correction, or, equivalently, –53 mV after correction. *Vm* measurements reported in the paper are corrected for a –13 mV liquid-liquid junction potential.

### Closed-loop visual environment

We presented visual stimuli on a panoramic LED array^77^ spanning 270° in azimuth and 81° in height, with ~1.875° pixel resolution^78^. The arena consisted of blue LEDs (BM-10B88MD, Betlux Electronics), covered by 7 sheets of blue gels (Tokyo Blue, Rosco) to reduce detection of light from the display by the microscope’s photomultiplier tubes. In patch-clamp electrophysiology experiments, we used the same LED display with 6 sheets of blue gels, 3 sheets of Clear-Shield film (Less EMF A1210–24) (that were electrically grounded) and one layer of stainless steel wire cloth (McMaster-Carr 85385T89) to minimize optical reflections between opposite sides of the display. In all experiments, we typically presented on the LED arena a 6-pixel (11°) wide bright blue vertical bar against a dark background. The bar rotated in closed loop with the fly’s yaw turns on an air-supported ball, thus simulating the movements of a distant landmark that can be used for orienting^78^. The rotations of the air-supported ball along the pitch, yaw and roll axes was detected via image analysis, using a custom-modified version of the open-source software FicTrac 2.0^79^. We modified the FicTrac 2.0 Python code to implement it within the Robot Operating System (ROS) platform, running it at 50 Hz during electrophysiology experiments and 80 Hz during imaging experiments. We illuminated the ball using 850 nm LEDs (OSRAM Platinum DRAGON, SFH 4235) guided by optical fibers (02–535, Edmund Optics). We imaged the ball using a Chameleon3 camera (CM3-U3–13Y3M, Teledyne FLIR) with an InfiniStix lens (144100, Infinity Photo-Optical). The lens was equipped with an 850/50 bandpass filter (84–778, Edmund Optics).

### Design and construction of the airflow delivery device

We designed an airflow delivery device based on a previously published apparatus^80^. The previous apparatus employed a single airflow outlet pointing at the fly, which could rotate to any arbitrary angle around the yaw axis. We modified this device to ensure that nothing visual changed in the field of view of the fly when the airflow direction needed to be altered. Our goal was to make it possible to present separable visual and airflow stimuli to the fly; with the original device, whenever one would change the airflow direction, the rotating nozzle necessarily created a concomitant moving visual stimulus. To solve these potential stimuli confounds, the new apparatus in this study separated the rotating outlet from the airflow delivery part, which took the form of a static disc surrounding the fly. The rotating outlet in the new device is a circular assembly consisting of a stepper motor driving a rotating nozzle. The nozzle delivers air, whose flow rate is regulated by a mass flow controller, to a subset of 36 circularly arrayed airflow channels. Depending on the stepper motor’s position, different airflow channels are engaged. Each of these 36 airflow channels was connected to a matching airflow channel in the airflow delivery disc that surrounded the fly via flexible plastic tubing, this is the part of the device featured in the figure schematics throughout the paper. The static disc consisted of 36 tubular airflow channels, spaced evenly at 10° increments, with each channel pointing at the fly from a different angle around the yaw axis. The connecting plastic tubes attached to the outer rim of the disc were gathered using soft Velcro tape and funneled away from the fly’s field of view. In summary, a motor-controlled rotating outlet induced airflow into a distributor manifold connected to the tubes, which passed through specific airflow channels in the disc and ultimately hit the fly’s body from a specific direction around the yaw axis.

The airflow disc surrounding the fly was sufficiently thin in the vertical dimension that the fly could still see the vertical blue bar on the LED display. Thus, we could change the airflow direction and visual experience of the fly completely independently. The ~1 m length of the plastic tubes does not significantly delay an airflow pulse from hitting the fly because air pressure changes can be considered as being transmitted instantaneously from one end of the tube to the other, for our purposes. Specifically, assuming a meter-long traveling distance, a airflow pulse from the rotating outlet would take ~2.9 ms to travel across the tube, governed by the speed of sound.

We should note that, in its current incarnation, this device has two main disadvantages. First, it is not well suited for rapidly switching between different odorants, because unlike with air pressure changes, odor molecules need to travel down the entire length of each plastic tube before reaching the fly; this process would take much longer than a few milliseconds. Second, the airflow direction is discretized by the presence of the 36 static tubes, introducing variation in airflow speed depending on the phase of the rotating outlet relative to the holes in the first static ring. Specifically, we found that when we made the nozzle opening exactly the diameter of one airflow channel, the air speed arriving at the fly varied up to ~50% depending on whether the nozzle was perfectly aligned with the channel or centered on the plastic midline between two channels, for example (data not shown). To combat this effect, we made the nozzle opening span two airflow channels, i.e., we made it 20° wide, which kept the proportion of the nozzle engaged with open air (within a channel) versus plastic more consistent independent of the nozzle’s position around the ring. This design feature minimized pressure changes that were contingent on the exact angle of airflow being delivered to the fly, while still maintaining a reasonable directional accuracy for the air stream.

#### 3D printing, part processing, and apparatus assembly

All custom parts were fabricated via 3D printing using VisiJet M3 Crystal material on a Projet MJP 3600 series 3D printer (3D Systems) at 30 μm resolution. We cleaned the printed parts using a multi-step procedure. First, the parts were incubated in an oven set to 65° C to melt and remove most of the wax support material. Afterward, the parts were sonicated in a mineral oil bath for at least one hour to dislodge the finer wax coating still attached to the plastic. The mineral oil residue was then removed by passing a jet of compressed air through all of the holes in the part. Afterwards, the parts were washed using water and dish soap (Dawn), rinsed with distilled water, and dried with compressed air. Each of the holes in the static airflow channels were manually tapped and fitted with a 1/16’’ brass hose barb (Clippard 12841) and the barbs on both parts (the airflow disc and the discretizing manifold on top of the rotating nozzle) were connected using soft plastic tubing (Tygon E-3603 via McMaster Carr, 5155T12). The rotating outlet component of the device was attached to an aluminum post and mounted on the same air table as the microscope. The airflow disc was mounted to the device that held the air-supported ball, which helped to ensure that the disc’s center was precisely located at the position in which the fly stood. Additionally, the airflow disc featured notches that nested the fly plates and ensured correct alignment of the fly’s body relative to the airflow disc during each experiment.

#### Airflow calibration

We calibrated the device by measuring the air speed at the center of the airflow disc with a hot wire anemometer (Climomaster Anemometer 6501-CE, Kanomax) fitted with an omnidirectional spherical probe (6543–2G, Kanomax). Using custom components, especially printed for alignment purposes, we positioned the spherical probe in the precise position that a fly would occupy during an experiment. We then manually rotated the nozzle so as to align it with the frontmost (0°) airflow channel. At this zero position, the 20° nozzle thus fully spanned the central airflow channel as well as ~5° each of the channels on either side of the frontmost channel. The airflow angles used in this paper were all multiples of 10° from this zero position, meaning that for all airflow stimuli we expect there to have been the same phase relationship between the nozzle opening and the airflow channels downstream of the nozzle. This fact makes it more likely that the air speed that the fly experienced was consistent across different delivery angles (because we noted changes in air speed that could occurred as a function of the phase relationship of the nozzle and the downstream, discrete, set of tubes). Indeed, we experimentally verified that the air speed was consistent at the position of the fly, with the exact air puff angles used in the paper, using the anemometer. We also validated the directionality of the air puffs delivered to the fly by visualizing CO_2_ gas (dry ice) with a laser sheet. We performed the dry ice visualization in a separate, but nominally identical, assembly.

### Simultaneous control of the airflow and bar stimuli

We used analog voltages––generated by the microcontroller that controlled the visual pattern on the LED display^77^––to control the angular position of the airflow stimulus as well as the mass flow controller that turned the airflow on and off. To control the angle of the airflow stimulus, the analog voltage controlled the position of a stepper motor that drove the rotating air outlet to new set points. To control the air speed, a voltage signal controlled the opening and closing of 2-SLPM mass flow controller (Alicat Scientific). The approach of having the LED-arena’s microcontroller also drive the airflow stimuli made onset and offset latencies of the air puffs more reliable and also aided temporal alignment between the visual and air-puff stimuli.

### Experimental protocol for presenting open-loop airflow and closed-loop visual stimuli

To solidify the visually mapping between the position of the blue bar on the LED display and the EPG bump position in the brain, all electrophysiology experiments and most imaging experiments began with a period of 5–10 minutes of the fly interacting with the bright blue bar in closed loop. This procedure was particularly important for electrophysiological data collection because we could not directly estimate the EPG phase in these experiments, and thus any changes in the mapping between the EPG phase and the visual cue would have manifested as multi-peaked heading tuning curves from single neurons. We could be less strict on this matter in two-photon imaging experiments because we were directly monitoring the EPG neuron population and could always estimate its phase in the brain, independent of the bar’s position on the LED arena. Following the acclimation period for establishing a robust EPG visual mapping, we began to present air puffs in open loop. We presented air puffs from twelve angles around the fly, in 30° increments, to fully and equally cover 360° of azimuthal space. The 0° direction, where air arrived from directly in front of the fly, was always included, which anchored the eleven other angles presented. Each open-loop air pulse lasted for 4 s with a 5 s inter-pulse interval. The air-puff angles were pseudorandomized in each block of twelve trials. We collected 3–5 blocks of trials per fly during imaging experiments, and 10 blocks during electrophysiology experiments. We used an airflow speed of 20 cm/s for all puffs. In this study, 0° represents the direction directly in front of the fly, and 180° represents the direction directly behind the fly. Negative angles represent air arriving to the fly’s egocentric left side, and positive angles other than 0° (front) and 180° (back) are to the egocentric right of the fly.

### Synchronized acquisition of behavior and physiology data

We recorded all experiment-related signals in the form of voltages using a Digidata 1440A I/O board and Axoscope software (Molecular Devices). The air-supported ball’s yaw, pitch, and roll angles, the control signals to and from the airflow stepper motor, the Alicat flow meter output, the angular position of the blue bar the LED display, the internal triggers of the FicTrac ball-tracking camera, and custom signals to identify trials epochs, were recorded at 10 kHz in all experiments. Signals specific to either calcium imaging or electrophysiology, such as the membrane potential, injected current, or triggers for two-photon imaging frame acquisition, were recorded in addition when applicable. Behavioral, electrophysiological, and calcium imaging signals updated at different rates and we used either the FicTrac camera’s triggers, or the two-photon imaging frame triggers, for careful temporal alignments to behavioral and brain-imaging signals, as required.

### Calcium imaging data analysis

#### Data processing

We registered raw time series of fluorescence images using either the rigid motion correction algorithm in the CaImAn software suite^81^ or a custom algorithm described previously^1,78^. Both registration approaches produced qualitatively similar results. When available, we used the tdTomato or jRGECO1a image for registration, otherwise, we used the jGCaMP7f signal directly. After registration, we drew regions of interest (ROIs) manually, in each z-plane separately, using custom software^41^ written in Python 3.8. We drew ROIs on fluorescence images averaged across an entire recording session, and our assignment of glomerular boundaries was aided by simultaneously viewing autocorrelation images using data from the entire session. When analyzing data in the protocerebral bridge, individual glomerulus identities were assigned by adhering to the following established anatomical principles^82^: (1) the entire protocerebral bridge should be composed of 18 distinct glomeruli, (2) PFN neurons do not innervate the pair of glomeruli closest to the midline, and (3) EPG neurons do not innervate the pair of glomeruli furthest from the midline. Out of a total of 12 collected bridge co-imaging datasets, we did not analyze 4 recordings in which more than one glomerulus was not visible, or whose identity could not be clearly established. When analyzing the ellipsoid body, we divided the structure into 16 evenly spaced radial sectors, whose boundaries and center were defined across every plane, as previously described^1,78^. Following previous conventions^1,78^, and to establish a consistent anatomical correspondence with the bridge and fan-shaped body, wedge 1 and wedge 16 of the ellipsoid body were defined, respectively, as the wedges immediately to the left and immediately to the right of the vertical bisector line at the bottom of the torus, when viewed from the posterior side of the head. When analyzing the fan-shaped body, we defined columns as follows. We first drew an outline of the entire structure. Then, the left and right edges of the structure were marked with angled lines. The vertex point at which the extension of these two angled lines met defined the entire angular extent of the fan-shaped body, which we then subdivided into 16 equally-spaced columns^1^. Column 1 of the fan-shaped body, which corresponds functionally to wedge 1 of the ellipsoid body, was defined as the leftmost fan-shaped body column when viewing the structure from the posterior side of the head. Noduli outlines were manually drawn and assigned as either left or right, using the same posterior view.

#### Fluorescence signal normalization

For each imaging volume and structure being imaged, we averaged together the signal from ROIs across z-planes, if they belonged to the same glomerulus, wedge, column or nodulus side, thus generating a final, unidimensional array of fluorescence intensity values for each structure at each time point. Each of these unidimensional arrays, corresponding to individual sector ROIs, were concatenated into a matrix where each data column corresponded to one sector and each row corresponded to a time point. Thus, the matrices of fluorescence values were composed of 18 data columns for the 18 glomeruli in the protocerebral bridge, 16 data columns for the 16 wedges of the ellipsoid body, 16 data columns for the 16 anatomical columns of the fan-shaped body, and 2 data columns for the 2 sides of the noduli. We report all imaging data from the ellipsoid body and fan-shaped body as ΔF/F, calculated as (F – F_min_) / (F_min_). In this equation, F corresponds to the raw fluorescence intensity measured in a given sector ROI (i.e. in each wedge or column) and F_min_ corresponds to the 5^th^ percentile of F values observed in that same sector ROI over the whole recording. When analyzing calcium imaging data from the protocerebral bridge, we normalized each glomerulus ROI to its own maximum and minimum F values because some glomeruli were much brighter or dimmer than others likely due to the expression levels of the fluorophore. Thus, the values of ΔF/F_max_-F_0_ that we report here for the protocerebral bridge correspond to (F – F_min_) / (F_max_ – F_min_), where F corresponds to the raw fluorescence signal in sector ROI, and F_min_ and F_max_ represent the 5^th^ and 95^th^ percentile of the F values from that same sector ROI over the entire recording. When analyzing the data from the noduli, we performed a z-score normalization over the raw fluorescence values, calculated separately for each of the left and right nodulus sector ROIs.

#### Aligning fluorescence readings with behavioral measurements

We matched behavioral (80 Hz) and imaging (4–8 Hz) measurements in the following manner. We determined the epochs corresponding to entire volumetric scan cycles using the voltage triggers output by the two-photon microscope, and we averaged the fluorescence values corresponding to each ROI across all z-slices containing them in the cycle. We averaged behavioral-related measurements, such as the visual stimulus’ position on the screen, over the time corresponding to an entire volumetric scan cycle as well. The behavioral signals are thus averaged over epochs of ~125–250 ms (corresponding to the imaging period given our scanning rates of 4–8 Hz).

#### Phase extraction

Columnar neurons in the central complex often express spatially localized calcium signals that are referred to as bumps of activity. Because space around the fly, 0°−360°, physically maps to positions in central complex structures, the position of the calcium bumps within a structure has an angular interpretation and thus is often referred to as the bump’s phase. We extracted the phase of calcium bumps in the ellipsoid body and fan-shaped body by taking a population vector average, as described previously^8,78,83^. We defined the sectors of the ellipsoid body and fan-shaped body to have their numerical values (1 to 16) match the known anatomical and functional correspondence across these two structures. Thus, when phase of bumps in these two structures matched, this meant that the angular signal carried by the two neuronal populations were functionally aligned in angular space, and phase differences across the ellipsoid body and fan-shaped body could be calculated through a simple angular subtraction as described in the Data processing subsection above. When imaging EPG neurons in the protocerebral bridge, the phase of the EPG heading estimate was extracted as previously described^78^. In brief, we took a Fourier transform of the sector ROI signal over the 16 glomeruli. The phase of the EPG bump in a given time point was then defined as the phase of the Fourier component with a wavelength of 8 glomeruli. This approach assumes that an invariable 8-glomerulus spacing exists between the peaks of the two EPG bumps in the bridge, and that, together, these two bumps encode a single, shared, heading angle estimate, which has proven to be a robust assumption.

#### Phase nulling

We phase-aligned EPG and PFN bumps in the protocerebral bridge using an algorithm described previously^78^. In brief, the sector ROI arrays from the protocerebral bridge for EPG and PFNa cells were first, separately, interpolated to 1/10th of a glomerulus resolution using a cubic spline. We then circularly rotated the vector at every time point so that the EPG phase was at the same position along the x axis. This EPG-determined rotation was then applied to the PFNa interpolated vector signal, allowing us to visualize the phase of the PFNa bumps in reference to that in the EPG system. It is only by employing this phase nulling analysis that we analyzed the phase of the PFNa bumps presented in this study. We found that we could not easily define the phase of the PFNa bumps otherwise, because they were not stably periodic nor always visible in calcium experiments.

### Electrophysiology data analysis

#### Data processing

We processed current-clamp recordings to extract each cell’s membrane potential (*Vm*), sodium-spike rate and 2–6 Hz power (due to calcium spiking). To make analysis of *Vm* convenient, we downsampled the raw 10kHz *Vm* traces (corrected for the liquid-liquid junction potential) to 1000 Hz. Afterwards, we median-filtered those traces using a kernel size of 40 ms to remove spikes. We detected sodium spike times by first bandpass filtering the raw 10kHz *Vm* traces in the range of 150–1000 Hz using an 8-pole Bessel filter. In this way, we selected for higher frequency events such as sodium spikes which are characterized by fast changes in the *Vm,* and we selected against lower frequency events such as subthreshold synaptic potentials. Second, we identified discrete events in the bandpass filtered trace based on crossing of a high threshold. Finally, we rejected all events with an inter-event interval of less than 6.66 ms. We labeled all events detected this way as sodium spikes, and visually inspected a subset of the events to ensure appropriate spike detection. We converted sodium-spike times to spike rate by convolving spike times with a sliding 1-second-wide Hann window. Sodium-spike amplitudes were often small––only a few millivolts or less––in these recordings, which is typical of recordings from somata of neurons in invertebrates. As a result, any spike-detection algorithm used is likely to have resulted in calling some non-spike events as spikes and to not have detect some number of genuine sodium spikes emitted by the cells. We do not view a potential, low rate of Type I and Type II errors as a substantive concern, however, for two reasons. First, our results depend on an overall estimate of spike rate and not precise spike times; some low level of spike misdetections might raise or lower the estimate rate over time, but this should not drastically impact the shape of the relevant tuning curves for our model. Second, empirically, the spike detection algorithm that we employed detected putative sodium spikes only extremely rarely when PFNa cells were hyperpolarized (e.g. Figures 3F-3G), arguing that we were not typically detecting subthreshold events, erroneously, as sodium action potentials.

We quantified calcium spikes by extracting membrane potential oscillation strength in the 2–6 Hz range. To extract oscillation strength, we downsampled raw *Vm* traces to 100 Hz and performed a fast Fourier transform on the traces (specgram function in Matplotlib; window size = 4 s; window step size = 20 ms). We averaged the calculated power of *Vm* frequencies in the range of 2–6 Hz and used this value as our metric for estimating the strength of T-type oscillations in PFNa neurons. To quantify the oscillation strength during 2 s periods of current injection, we first conditioned the current-injection *Vm* traces as follows. We defined two 600-ms time segments. One segment started 550 ms before the start of the current injection pulse and the second segment started 50 ms before the end of the current injection pulse. We then replaced the actual *Vm* sample points in both segments with a linear interpolation between the first and last sample point within the time segment. We then took the fast Fourier transform of the *Vm* traces and quantified the power in the 2–6 Hz band, using a window size of 2 s and window step size of 0.01 s. The linear interpolation of the *Vm* trace that we performed at the onset and offset of the current pulse prevented artifactual frequencies from appearing to have high power in the Fourier transform analysis simply due to the large *Vm* step-like changes that necessarily occur at the start and end of the current injection pulse.

To align behavioral readings with electrophysiological measurements, the sample points from the FicTrac camera were upsampled to 1000 Hz, using linear interpolation, to match the analyzed membrane potential signal, which was downsampled to 1000 Hz as described above.

#### *Estimation of baseline* Vm

PFNa neurons express large calcium spikes, alongside large synaptic potentials, even when very hyperpolarized, which makes defining a resting *Vm* difficult for these cells. Thus, instead of estimating a resting *Vm* for PFNa cells, we instead defined the baseline *Vm* as the minimal value in the heading tuning curve calculated for each cell (mean: −64 mV, standard deviation: 4.8 mV, range: 19.9 mV). This baseline *Vm* value was typically stable during our recordings, which usually lasted 45 to 60 minutes. Whereas the baseline membrane potential varied substantively across cells (Figures S3A and S3B), we found that the peak-to-minimum membrane potential amplitude of the heading tuning curve (mean: 13.7 mV, standard deviation: 2.9 mV), and the sodium spike rate at the minimum value of the heading tuning curve (mean: 0 spikes/s, standard deviation: 0.1 spikes/s) was more consistent across cells. Given this observation, membrane potential data was combined across cells by subtracting the baseline membrane potential from each cell. We compare tuning curves with and without normalization in Figure S3. Spike rate data were combined across cells without any such normalization.

#### Estimation of heading, airflow direction and conjunctive tuning curves

For each PFNa cell, we generated *Vm*, spike rate, and oscillation strength tuning curves as a function of heading and airflow angle. We generated single-variable tuning curves against these two variables, and we also generated conjunctive, two-variable (heat map) tuning curves against the two variables. For heading tuning curves, we binned the time series of bar positions on the LED display into 10° bins, and we averaged the *Vm*, spike rate or oscillation strength in each bin. We required that a bin included at least 2 s worth of sample points for its average to be calculated. We also required that the fly not be standing (forward speed > 0.5 mm/s) for sample points to be included in the tuning curve. We calculated the preferred heading direction of a given cell’s tuning curve by finding the angular shift of the curve that produced the highest correlation between the actual tuning curve and a normalized cosine function. We calculated tuning curves to air puffs by averaging the *Vm*, spike rate, or oscillation strength in the 10 air-puff trials for each of 12 air puff directions presented in a 2 s window, starting 0.5 s after the onset of the air puff. To estimate conjunctive tuning to heading and airflow, we split the data into 18 (20° heading) x 12 (45° airflow direction) bins. We required that at least 0.5 s of data populate each bin in order to calculate a mean value. After calculating the conjunctive tuning heat-map for each cell separately, we generated combined heat maps across cells after centering each single-cell heat map on the preferred heading direction of a given cell.

### Model construction

Membrane potential data over 12 egocentric airflow directions, W, and 18 relative heading directions, *H* (the heading direction relative to the preferred heading direction for a particular neuron), averaged over the recorded PFNa left and right neurons, were fit using the form $V_{m}=a_{o}\;+\;a_{H}\;\text{cos}\left(H\right)+a_{W}\;\text{cos }\left(W\pm \text{π}/4\right)$, with free parameters *a*_*o*_, *a*_*H*_ and *a*_*W*_, and the ± applied to left or right neurons, respectively. This 3-parameter fit of 216 data points for each case (left/right) explained 93.7% (left) and 90.9% (right) of the variance of the data.

Firing rates for sodium spikes were fit to the equation $r={\left({\left[b_{H}\;\text{cos}\left(H\right)+b_{W}\;\text{cos }\left(W\pm \text{π}/4\right)\right]}_{+}\right)}^{2}$, with *b*_*H*_ and *b*_*W*_ free parameters and ${\left[x\right]}_{+}=x$ for *x* > 0 and 0 otherwise. This two-parameter fit of 216 data points (as above) explained 85.9% and 85.7% of the variance for the left/right PFNa neurons, respectively. We fit the oscillation power for these data to $p=c_{0}\;+{\left({\left[c_{H}\;\text{cos}\left(H\right)+c_{W}\text{ cos}\left(W\pm \text{π}/4\right)\right]}_{-}\right)}^{2}$, with *c*_0_, *c*_*W*_, and *c*_*H*_ free parameters and ${\left[x\right]}_{-}=x$ for *x* < 0 and 0 otherwise. This three-parameter fit of 216 data points explained 71.4% and 77.1% of the variance for the left/right PFNa neurons.

For the mathematical model of the full response discussed in the text, we assumed that the plus and minus rectifications discussed in the previous paragraph could be combined, when modeling the output of a PFNa neuron across all angles. This means that we assumed that the proportionality constant relating output due to calcium spikes to output due to sodium spikes is such that the total output can be expressed as ${\left(b_{H}\;\text{cos}\left(H\right)+b_{W}\;\text{cos }\left(W\pm \text{π}/4\right)\right)}^{2}$, with no rectification as a consequence of summing sodium and calcium spike contribution. Summing the left and right contributions and accounting for the ±45° anatomical shifts of the PFNa projections from the protocerebral bridge to the fan-shaped body gives the total signal from this similarly tuned pair as

$$\begin{array}{c} {\left(b_{H}\cos\left(H-\frac{\text{π}}{4}\right)+b_{W}\cos\left(W-\frac{\text{π}}{4}\right)\right)}^{2}+{\left(b_{H}\cos\left(H-\frac{\text{π}}{4}\right)+b_{W}\cos\left(W-\frac{\text{π}}{4}\right)\right)}^{2}\\ =b_{H}{}^{2}\left(\cos{\left(H-\frac{\text{π}}{4}\right)}^{2}+\cos{\left(H+\frac{\text{π}}{4}\right)}^{2}\right)+b_{W}{}^{2}\left(\cos{\left(W+\frac{\text{π}}{4}\right)}^{2}+\cos{\left(W-\frac{\text{π}}{4}\right)}^{2}\right)\\ +2b_{H}b_{W}\left(\cos\left(H-\frac{\text{π}}{4}\right)\cos\left(W+\frac{\text{π}}{4}\right)+\cos\left(H+\frac{\text{π}}{4}\right)\cos\left(W-\frac{\text{π}}{4}\right)\right). \end{array}$$

Because $\text{cos}\left(x-\text{π}/4\right)=\text{sin}\left(x+\text{π}/4\right)$, and the sum of squared sines and cosines is 1, the first line on the right of the equal sign above equals $b_{H}{}^{2}+b_{W}{}^{2}$. Using the sum-of-angles identity, $\cos\left(H\bar{+}\text{π}/4\right)\cos\left(W\pm \text{π}/4\right)=\left(\cos\left(W+H\right)+\cos\left(W-H+\text{π}/2\right)\right)/2$, and noting that $\text{cos}\left(x+\text{π}/2\right)+\text{cos}\left(x+\text{π}/2\right)=0$, the last expression above becomes $2b_{H}b_{W}\text{cos}\left(W+H\right)$. Putting this all together, we find that the total output of this pair of PFNa neurons is $b_{H}{}^{2}\;+\;b_{W}{}^{2}\;+\;2b_{H}b_{W}\text{cos}\left(W+H\right)$, as given in the text. The output across the full population of PFNa neurons, parameterized by their preferred heading angle *θ* is then a phasor representing a vector of unit length pointing in the allocentric direction of the airflow.

### Immunohistochemistry

We dissected adult fly brains and fixed them in 2% paraformaldehyde for 55 minutes in 24-well crystallization plates (Cryschem M Plate, Hampton Research). We washed the brains 3x for 20 minutes each in phosphate-buffered saline containing 0.5% Triton-X (PBST), then blocked them with 5% normal goat serum (NGS, sourced from Gibco). For immunostaining, we used a primary antibody solution consisting of 1:1000 chicken anti-GFP (600–901-215, Rockland Immunochemicals),1:30 mouse anti-bruchpilot (nc82, Developmental Studies Hybridoma Bank), and 5% NGS diluted in PBST. We nutated the brain samples in primary antibody solution for 4 h at room temperature, followed by an overnight rocking incubation at 4°C. Afterwards, we washed the brains 3x for 20 minutes each in PBST, then incubated them in a secondary antibody solution, composed of 1:800 goat anti-chicken Alexa Fluor 488 (A11039, ThermoFisher Scientific), 1:200 goat anti-mouse Alexa Fluor 594 (A11032, ThermoFisher Scientific), and 5% NGS in PBST. We nutated the brains for 4 h at room temperature, then incubated them overnight at 4°C. Afterwards, we washed the brains 3x for 20 minutes each in PBST, performed a final wash in phosphate-buffered saline, and mounted the brains onto glass slides in 8 μl of FocusClear (CelExplorer) with the posterior side up (i.e. the posterior end of the brain faced the coverslip). We imaged the mounted brains using an upright Zeiss LSM 780 confocal microscope fitted with a 20× 0.8NA air objective (Plan-Apochromat 20x/0.8, Zeiss). Each stack of images consisted of 80–120 optical sections spaced ~1 μm apart.

### Analysis of open-source RNA sequencing dataset

The data in Figure 4B were obtained from an open-source RNA sequencing dataset by Davis et al, 2020^14^, available in the NlH’s Gene Expression Omnibus (GEO)^84,85^ under accession number GSE116969. The specific dataset shown here is GSE116969_dataTable4.genes_x_cells_TPM.coding_genes_QCpass, which reports the cell type-mean abundance of protein coding genes in quality control-passed samples. No additional processing was performed on the reported abundances.

### Analysis of open-source hemibrain connectome dataset

All electron microscopy-based analysis in this study was performed using the hemibrain connectome^59^ dataset v1.2.1 and neuPrint-python^86^ v0.4.15. Skeleton renderings were performed using the NAVis python library v1.3.1.

### Statistics

Details on statistical testing, as well as exact p-values, are presented below in Table 3. Throughout the paper, we used a circular mean function implemented after Fisher and Lee, 1983^87^. We performed t-tests using the scipy.stats package^88^ and we performed Watson-Williams tests using the pycircstat package, a Python implementation of CircStat^89^, a toolbox for directional statistics in MATLAB.

## Supplementary Material

Supplement 1

## Figures and Tables

**Table 1.** Experimental crosses.

Figure	Parent 1	Parent 2
Figure 2B, 2C, 2D, 2E; Figure S2A, S2F, S2H; Figure 5F;	w+; LexAop-RGECO1a in su(Hw)attp5; UAS-jGCaMP7f in VK00005	w+; 60D05-LexA in attp40; 30E10-Gal4 in attp2
Figure 2F; Figure S2B, S2C, S2G	w+; ; UAS-jGCaMP7f in VK00005	30E10-Gal4 in attp2
Figure 3B, 3C, 3E, 3F, 3G, 3H, 3I; Figure S2I; Figure S3A, S3B, S3C, S3D, S3E, S3F, Figure S4A, S4B, S4D, S4G, S4J; Figure 5C, 5D, 5E	w+; UAS-2xeGFP (in Chr 2)	SS02255 (VT016D01-p65ADZp in attp40; VT016114-ZpGdbd in attp2)
Figure 4C	Ca-α1T^GFP^	Ca-α1T^GFP^
Figure 4D, 4G; Figure S4G, S4I	w+; attp40; UAS-Dcr2 (in Chr 3)	w+; UAS-2xeGFP (in Chr 2); 30E10-Gal4 in attp2
Figure 4G; Figure S4H; S4J	w+; TRiP.HMS.01948 in attp40; UAS-Dcr2 (in Chr 3)	w+; UAS-2xeGFP (in Chr 2); 30E10-Gal4 in attp2
Figure 4E, 4F	w+; attp40; UAS-Dcr2 (in Chr 3)	30E10-Gal4 in attp2, UAS-jGCaMP7f in VK00005
Figure 4H, 4I	w+; TRiP.HMS01948 in attp40	30E10-Gal4 in attp2, UAS-jGCaMP7f in VK00005
Figure 6D, 6E, 6F, 6G	w+; 27F02-LexA in attp40, LexAop-syt- jGCaMP8s in VK00022; UAS-CsChrimson-tdTomato in attp2	w+; 12E04-LexA in attp40; 41H07-Gal4 in attp2
Figure 6E, 6F, 6G	w+; 27F02-LexA in attp40, LexAop-syt- jGCaMP8s in VK00022; UAS-CsChrimson-tdTomato in attp2	w+; 12E04-LexA in attp40; VT056655-Gal4 in attp2
Figure 6E, 6F, 6G	w+; 27F02-LexA in attp40, LexAop-syt- jGCaMP8s in VK00022; UAS-CsChrimson-tdTomato in attp2	w+; 12E04-LexA in attp40; empty Gal4 in attp2
Figure 6I, 6J, 6K, 6L	w+; 27F02-LexA in attp40, LexAop-syt- jGCaMP8s in VK00022; UAS-GtACR-HA in VK00005	w+; 12E04-LexA in attp40; 41H07-Gal4 in attp2
Figure 6J, 6K, 6L	w+; 27F02-LexA in attp40, LexAop-syt- jGCaMP8s in VK00022; UAS-GtACR-HA in VK00005	w+; 12E04-LexA in attp40; VT056655-Gal4 in attp2
Figure 6J, 6K, 6L	w+; 27F02-LexA in attp40, LexAop-syt- jGCaMP8s in VK00022; UAS-GtACR-HA in VK00005	w+; 12E04-LexA in attp40; empty Gal4 in attp2
Figure 6N, 6O, 6P, 6Q	Ca-α1T^del^; 27F02-LexA in attp40, LexAop-syt- jGCaMP8s in VK00022; UAS-GtACR-HA in VK00005	Ca-α1T^Δ135^; 12E04-LexA in attp40; 41H07-Gal4 in attp2
Figure 6O, 6P, 6Q	Ca-α1T^del^; 27F02-LexA in attp40, LexAop-syt- jGCaMP8s in VK00022; UAS-GtACR-HA in VK00005	w+; 12E04-LexA in attp40; 41H07-Gal4 in attp2
Figure S1A, S1B, S1C, S1D, S1E, S1F, S1G, S1H, S1I; Figure S6B, S6C, S6D, S6E	LexAop-jGCaMP7f in su(Hw)attp8; ; UAS-jRGECO1a in VK00005	w+; 12E04-LexA in attp40; 60D05-Gal4 in attp2
Figure S2D, S2E, S2G	w+; ; UAS-jGCaMP7f in VK00005	SS47432 (VT046049-p65ADZp in attp40; VT024603-ZpGdbd in attp2)
Figure S2G	UAS-GFlamp1 in VK00005	w+; SS02255 (VT016D01-p65ADZp in attp40; VT016114-ZpGdbd in attp2)
Figure S4K	w+; UAS-2xeGFP; UAS-GtACR-HA in VK00005	w+; SS02255 (VT016D01-p65ADZp in attp40; VT016114-ZpGdbd in attp2)

**Table 2.** Stock sources.

Stock	Source	Identifier
UAS-jGCaMP7f in VK00005	Bloomington Drosophila Stock Center	RRID: BDSC_79031
LexAop-jGCaMP7f in su(Hw)attp8	Bloomington Drosophila Stock Center	RRID: BDSC_80910
LexAop-syt-jGCaMP8s in VK00022	This study, gift from Cheng Lyu and Stephen Thornquist	N/A
UAS-jRGECO1a in VK00005	Bloomington Drosophila Stock Center	RRID: BDSC_63794
LexAop2-jRGECO1a in su(Hw)attp5	Bloomington Drosophila Stock Center	RRID: BDSC_64426
UAS-GtACR1-HA in VK00005	Vanessa Ruta lab via Vikram Vijayan^71^	N/A
UAS-CsChrimson-tdTomato in attp2	David Anderson and Barret Pfeiffer via Stephen Thornquist^72^	N/A
UAS-myr-tdTomato in attp40	Bloomington Drosophila Stock Center	RRID: BDSC_32222
UAS-GFlamp1 in VK00005	This study, gift from Stephen Thornquist	N/A
UAS-2xeGFP (chromosome 2)	Michael Dickinson lab	N/A
TRiP.HMS01948 in attp40	Bloomington Drosophila Stock Center	RRID: BDSC_39029
attP40	Bloomington Drosophila Stock Center	RRID: BDSC_36304
UAS-Dcr-2 (chromosome 3)	Bloomington Drosophila Stock Center	RRID: BDSC_24651
Ca-α1T^GFP^	Bloomington Drosophila Stock Center	RRID: BDSC_68202
Ca-α1T^Δ135^	Bloomington Drosophila Stock Center	RRID: BDSC_68200
Ca-α1T^del^	Bloomington Drosophila Stock Center	RRID: BDSC_51994
60D05-Gal4 in attp2	Bloomington Drosophila Stock Center	RRID: BDSC_39247
30E10-Gal4 in attp2	Bloomington Drosophila Stock Center	RRID: BDSC_49638
41H07-Gal4 in attp2	Bloomington Drosophila Stock Center	RRID: BDSC_46241
VT056655-Gal4 in attp2	Janelia Research Campus	N/A
Empty-Gal4	Bloomington Drosophila Stock Center	RRID: BDSC_68384
SS02255 (VT016D01-p65ADZp ß VT016114-ZpGdbd)	Bloomington Drosophila Stock Center	RRID: BDSC_75923
SS047432 (VT046049-p65ADZp ß VT024603-ZpGdbd)	Bloomington Drosophila Stock Center	RRID: BDSC_86716
60D05-LexA in attp40	Bloomington Drosophila Stock Center	RRID: BDSC_52867
27F02-LexA in attp40	Bloomington Drosophila Stock Center	RRID: BDSC_52748
12E04-LexA in attp40	Bloomington Drosophila Stock Center	RRID: BDSC_52447

**Table 3.** Statistical testing.

	Groups compared	Test	Null hypothesis	P-value (P<0.05 in bold)
4D, 4G	Mean 2–6 Hz power below −60 mV for attp40 (*panel 4D, left side*) vs Ca-α1T RNAi (*panel 4G, left side*)	t-test, two-sided, means of two independent samples	Samples have equal means	**8.88×10** ^**−4**^
4F, 4I	Absolute R-L noduli z score at −120° airflow for attp40 (*panel 4F, left side*) vs Ca-α1T RNAi (*panel 4I, left side*)	t-test, two-sided, means of two independent samples	Samples have equal means	**1.13×10** ^**−2**^
4F, 4I	Absolute R/L noduli z score at 120° airflow for attp40 (*panel 3F, left side*) vs Ca-α1T RNAi (*panel 3G, left side*)	t-test, two-sided, means of two independent samples	Samples have equal means	**1.62×10** ^**−2**^
6G	Empty-Gal4 vs 41H07-Gal4	t-test, two-sided, means of two independent samples	Samples have equal means	**6.21×10** ^**−4**^
6G	Empty-Gal4 vs VT056655-Gal4	t-test, two-sided, means of two independent samples	Samples have equal means	**2.44×10** ^**−2**^
6L	Empty-Gal4 vs 41H07-Gal4	Watson-Williams test for equality of the means of two or more samples of circular data	Samples have equal means	**8.97×10** ^**−7**^
6L	Empty-Gal4 vs VT056655-Gal4	Watson-Williams test for equality of the means of two or more samples of circular data	Samples have equal means	**8.66×10** ^**−7**^
6Q	Heterozygous (+/del) vs null (del/Δ135)	Watson-Williams test for equality of the means of two or more samples of circular data	Samples have equal means	**5.95×10** ^**−9**^
S1E	No airflow vs airflow	t-test, two-sided, means of two paired samples	Samples have equal means	0.725
S1G	No airflow vs airflow	t-test, two-sided, means of two paired samples	Samples have equal means	0.906
S1H	No airflow vs airflow	t-test, two-sided, means of two paired samples	Samples have equal means	0.290
S4H, S4I	Mean 2–6 Hz power for current injections below - 20 pA for attp40 (*panel* S4H, *left side*) vs Ca-α1T RNAi (*panel* S4I, *left side*)	t-test, two-sided, means of two independent samples	Samples have equal means	**1.91×10** ^**−5**^

## References

[1] Lyu C., Abbott L.F., and Maimon G. (2022). Building an allocentric travelling direction signal via vector computation. Nature 601, 92–97. 10.1038/s41586-021-04067-0. 34912112 PMC11104186

[2] Wittmann T., and Schwegler H. (1995). Path integration - a network model. Biological Cybernetics.

[3] Stone T., Webb B., Adden A., Weddig N.B., Honkanen A., Templin R., Wcislo W., Scimeca L., Warrant E., and Heinze S. (2017). An Anatomically Constrained Model for Path Integration in the Bee Brain. Current Biology 27, 3069–3085.e11. 10.1016/j.cub.2017.08.052. 28988858 PMC6196076

[4] Hulse B.K., Haberkern H., Franconville R., Turner-Evans D.B., Takemura S., Wolff T., Noorman M., Dreher M., Dan C., Parekh R., (2021). A connectome of the *Drosophila* central complex reveals network motifs suitable for flexible navigation and context-dependent action selection. eLife. 10.7554/eLife.66039. PMC9477501 34696823

[5] Lu J., Behbahani A.H., Hamburg L., Westeinde E.A., Dawson P.M., Lyu C., Maimon G., Dickinson M.H., Druckmann S., and Wilson R.I. (2022). Transforming representations of movement from body- to world-centric space. Nature 601, 98–104. 10.1038/s41586-021-04191-x. 34912123 PMC10759448

[6] Steriade M., and Llinás R.R. (1988). The functional states of the thalamus and the associated neuronal interplay. Physiological Reviews 68, 649–742. 10.1152/physrev.1988.68.3.649. 2839857

[7] McCormick D.A., and Bal T. (1997). SLEEP AND AROUSAL: Thalamocortical Mechanisms. Annu. Rev. Neurosci. 20, 185–215. 10.1146/annurev.neuro.20.1.185. 9056712

[8] Seelig J.D., and Jayaraman V. (2015). Neural dynamics for landmark orientation and angular path integration. Nature 521, 186–191. 10.1038/nature14446. 25971509 PMC4704792

[9] Green J., Vijayan V., Mussells Pires P., Adachi A., and Maimon G. (2019). A neural heading estimate is compared with an internal goal to guide oriented navigation. Nat Neurosci 22, 1460–1468. 10.1038/s41593-019-0444-x. 31332373 PMC7688015

[10] Green J., and Maimon G. (2018). Building a heading signal from anatomically defined neuron types in the Drosophila central complex. Current Opinion in Neurobiology 52, 156–164. 10.1016/j.conb.2018.06.010. 30029143 PMC6320682

[11] Hartmann G., and Wehner R. (1995). The ant’s path integration system: a neural architecture. Biological Cybernetics 73, 483–497.

[12] Currier T.A., Matheson A.M., and Nagel K.I. (2020). Encoding and control of orientation to airflow by a set of *Drosophila* fan-shaped body neurons. eLife 9, e61510. 10.7554/eLife.61510. 33377868 PMC7793622

[13] Okubo T.S., Patella P., D’Alessandro I., and Wilson R.I. (2020). A Neural Network for Wind-Guided Compass Navigation. Neuron 107, 924–940.e18. 10.1016/j.neuron.2020.06.022. 32681825 PMC7507644

[14] Davis F.P., Nern A., Picard S., Reiser M.B., Rubin G.M., Eddy S.R., and Henry G.L. (2020). A genetic, genomic, and computational resource for exploring neural circuit function. eLife 9, e50901. 10.7554/eLife.50901. 31939737 PMC7034979

[15] Jeong K., Lee S., Seo H., Oh Y., Jang D., Choe J., Kim D., Lee J.-H., and Jones W.D. (2015). Ca-α1T, a fly T-type Ca2+ channel, negatively modulates sleep. Sci Rep 5, 17893. 10.1038/srep17893. 26647714 PMC4673464

[16] Ni J.-Q., Zhou R., Czech B., Liu L.-P., Holderbaum L., Yang-Zhou D., Shim H.-S., Tao R., Handler D., Karpowicz P., (2011). A genome-scale shRNA resource for transgenic RNAi in Drosophila. Nat Methods 8, 405–407. 10.1038/nmeth.1592. 21460824 PMC3489273

[17] Klapoetke N.C., Murata Y., Kim S.S., Pulver S.R., Birdsey-Benson A., Cho Y.K., Morimoto T.K., Chuong A.S., Carpenter E.J., Tian Z., (2014). Independent optical excitation of distinct neural populations. Nat Methods 11, 338–346. 10.1038/nmeth.2836. 24509633 PMC3943671

[18] Zhang Y., Rózsa M., Liang Y., Bushey D., Wei Z., Zheng J., Reep D., Broussard G.J., Tsang A., Tsegaye G., (2023). Fast and sensitive GCaMP calcium indicators for imaging neural populations. Nature 615, 884–891. 10.1038/s41586-023-05828-9. 36922596 PMC10060165

[19] Govorunova E.G., Sineshchekov O.A., Janz R., Liu X., and Spudich J.L. (2015). Natural light-gated anion channels: A family of microbial rhodopsins for advanced optogenetics. Science 349, 647–650. 10.1126/science.aaa7484. 26113638 PMC4764398

[20] Ryglewski S., Lance K., Levine R.B., and Duch C. (2012). Ca v 2 channels mediate low and high voltage-activated calcium currents in *Drosophila* motoneurons: Novel roles for Dmca1A and DmαG calcium channels. The Journal of Physiology 590, 809–825. 10.1113/jphysiol.2011.222836. 22183725 PMC3381312

[21] Bell W.J., and Kramer E. (1979). Search and anemotactic orientation of cockroaches. Journal of Insect Physiology 25, 631–640. 10.1016/0022-1910(79)90112-4.

[22] Budick S.A., and Dickinson M.H. (2006). Free-flight responses of *Drosophila melanogaster* to attractive odors. Journal of Experimental Biology 209, 3001–3017. 10.1242/jeb.02305. 16857884

[23] Böhm H., Heinzel H.-G., Scharstein H., and Wendler G. (1991). The course control system of beetles walking in an air-current field. J Comp Physiol A 169. 10.1007/BF00194896.

[24] Dacke M., Bell A.T.A., Foster J.J., Baird E.J., Strube-Bloss M.F., Byrne M.J., and el Jundi B. (2019). Multimodal cue integration in the dung beetle compass. Proc. Natl. Acad. Sci. U.S.A. 116, 14248–14253. 10.1073/pnas.1904308116. 31235569 PMC6628800

[25] Kennedy J.S. (1951). The migration of the Desert Locust (Schistocerca gregaria Forsk.) I. The behaviour of swarms. II. A theory of long-range migrations. PHILOSOPHICAL TRANSACTIONS OF THE ROYAL SOCIETY OF LONDON 235, 163–290. 24541037 10.1098/rstb.1951.0003

[26] Linsenmair K.E. (1972). Anemomenotactic Orientation in Beetles and Scorpions. Animal Orientation and Navigation.

[27] Marsh D., Kennedy J.S., and Ludlow A.R. (1978). An analysis of anemotactic zigzagging flight in male moths stimulated by pheromone. Physiol Entomol 3, 221–240. 10.1111/j.1365-3032.1978.tb00152.x.

[28] Müller M., and Wehner R. (2007). Wind and sky as compass cues in desert ant navigation. Naturwissenschaften 94, 589–594. 10.1007/s00114-007-0232-4. 17361400

[29] Leitch K.J., Ponce F.V., Dickson W.B., van Breugel F., and Dickinson M.H. (2021). The long-distance flight behavior of *Drosophila* supports an agent-based model for wind-assisted dispersal in insects. Proc. Natl. Acad. Sci. U.S.A. 118, e2013342118. 10.1073/pnas.2013342118. 33879607 PMC8092610

[30] Matheson A.M.M., Lanz A.J., Medina A.M., Licata A.M., Currier T.A., Syed M.H., and Nagel K.I. (2022). A neural circuit for wind-guided olfactory navigation. Nat Commun 13, 4613. 10.1038/s41467-022-32247-7. 35941114 PMC9360402

[31] Suver M.P., Matheson A.M.M., Sarkar S., Damiata M., Schoppik D., and Nagel K.I. (2019). Encoding of Wind Direction by Central Neurons in *Drosophila*. Neuron 102, 828–842.e7. 10.1016/j.neuron.2019.03.012. 30948249 PMC6533146

[32] Patella P., and Wilson R.I. (2018). Functional Maps of Mechanosensory Features in the *Drosophila* Brain. Current Biology 28, 1189–1203.e5. 10.1016/j.cub.2018.02.074. 29657118 PMC5952606

[33] Eberl D.F., Hardy R.W., and Kernan M.J. (2000). Genetically Similar Transduction Mechanisms for Touch and Hearing in *Dro*sophila. J. Neurosci. 20, 5981–5988. 10.1523/JNEUROSCI.20-16-05981.2000. 10934246 PMC6772586

[34] Yorozu S., Wong A., Fischer B.J., Dankert H., Kernan M.J., Kamikouchi A., Ito K., and Anderson D.J. (2009). Distinct sensory representations of wind and near-field sound in the *Drosophila* brain. Nature 458, 201–205. 10.1038/nature07843. 19279637 PMC2755041

[35] Gallio M., Ofstad T.A., Macpherson L.J., Wang J.W., and Zuker C.S. (2011). The Coding of Temperature in the Drosophila Brain. Cell 144, 614–624. 10.1016/j.cell.2011.01.028. 21335241 PMC3336488

[36] Barbagallo B., and Garrity P.A. (2015). Temperature sensation in *Drosophila*. Current Opinion in Neurobiology 34, 8–13. 10.1016/j.conb.2015.01.002. 25616212 PMC4508239

[37] Morley E.L., Steinmann T., Casas J., and Robert D. (2012). Directional cues in *Drosophila melanogaster* audition: structure of acoustic flow and inter-antennal velocity differences. Journal of Experimental Biology 215, 2405–2413. 10.1242/jeb.068940. 22723479

[38] Göpfert M.C., and Robert D. (2002). Auditory mechanics of *Drosophila melanogaster*. Journal of Experimental Biology 205, 1199–1208. 11948197 10.1242/jeb.205.9.1199

[39] Kamikouchi A., Inagaki H.K., Effertz T., Hendrich O., Fiala A., Göpfert M.C., and Ito K. (2009). The neural basis of *Drosophila* gravity-sensing and hearing. Nature 458, 165–171. 10.1038/nature07810. 19279630

[40] Sun Y., Liu L., Ben-Shahar Y., Jacobs J.S., Eberl D.F., and Welsh M.J. (2009). TRPA channels distinguish gravity sensing from hearing in Johnston’s organ. Proc. Natl. Acad. Sci. U.S.A. 106, 13606–13611. 10.1073/pnas.0906377106. 19666538 PMC2717111

[41] Pires P.M., Abbott L.F., and Maimon G. (2022). Converting an allocentric goal into an egocentric steering signal (bioRxiv Neuroscience) 10.1101/2022.11.10.516026. PMC10881393 38326612

[42] Westeinde E.A., Kellogg E., Dawson P.M., Lu J., Hamburg L., Midler B., Druckmann S., and Wilson R.I. (2022). Transforming a head direction signal into a goal-oriented steering command (bioRxiv Neuroscience) 10.1101/2022.11.10.516039. PMC10881397 38326621

[43] Dan C., Kappagantula R., Hulse B.K., Jayaraman V., and Hermundstad A.M. (2021). Flexible control of behavioral variability mediated by an internal representation of head direction (Neuroscience) 10.1101/2021.08.18.456004.

[44] Harris K.P., and Littleton J.T. (2015). Transmission, Development, and Plasticity of Synapses. Genetics 201, 345–375. 10.1534/genetics.115.176529. 26447126 PMC4596655

[45] Van Vactor D., and Sigrist S.J. (2017). Presynaptic morphogenesis, active zone organization and structural plasticity in Drosophila. Current Opinion in Neurobiology 43, 119–129. 10.1016/j.conb.2017.03.003. 28388491 PMC5501089

[46] Iniguez J., Schutte S.S., and O’Dowd D.K. (2013). Ca v 3-type α1T calcium channels mediate transient calcium currents that regulate repetitive firing in *Drosophila* antennal lobe PNs. Journal of Neurophysiology 110, 1490–1496. 10.1152/jn.00368.2013. 23864373 PMC4042424

[47] Neher E. (1998). Vesicle Pools and Ca2+ Microdomains: New Tools for Understanding Their Roles in Neurotransmitter Release. Neuron 20, 389–399. 10.1016/S0896-6273(00)80983-6. 9539117

[48] Balkowiec A., and Katz D.M. (2002). Cellular Mechanisms Regulating Activity-Dependent Release of Native Brain-Derived Neurotrophic Factor from Hippocampal Neurons. J. Neurosci. 22, 10399–10407. 10.1523/JNEUROSCI.22-23-10399.2002. 12451139 PMC6758764

[49] Mansvelder H.D., and Kits K.S. (2000). Calcium channels and the release of large dense core vesicles from neuroendocrine cells: spatial organization and functional coupling. Progress in Neurobiology 62, 427–441. 10.1016/S0301-0082(00)00003-4. 10856612

[50] De Koninck P., and Schulman H. (1998). Sensitivity of CaM Kinase II to the Frequency of Ca 2+ Oscillations. Science 279, 227–230. 10.1126/science.279.5348.227. 9422695

[51] Magee J.C., and Grienberger C. (2020). Synaptic Plasticity Forms and Functions. Annu. Rev. Neurosci. 43, 95–117. 10.1146/annurev-neuro-090919-022842. 32075520

[52] Xiao K., Li Y., Chitwood R.A., and Magee J.C. (2023). A critical role for CaMKII in behavioral timescale synaptic plasticity in hippocampal CA1 pyramidal neurons. bioRxiv. 10.1126/sciadv.adi3088 PMC10482326 37672577

[53] Llinás R., and Yarom Y. (1981). Properties and distribution of ionic conductances generating electroresponsiveness of mammalian inferior olivary neurones in vitro. The Journal of Physiology 315, 569–584. 7310722 10.1113/jphysiol.1981.sp013764 PMC1249399

[54] Lee J., Kim D., and Shin H.-S. (2004). Lack of delta waves and sleep disturbances during non-rapid eye movement sleep in mice lacking α1G-subunit of T-type calcium channels. Proceedings of the National Academy of Sciences 101, 18195–18199. 10.1073/pnas.0408089101 PMC539778 15601764

[55] Cheong E., and Shin H.-S. (2013). T-Type Ca 2+ Channels in Normal and Abnormal Brain Functions. Physiological Reviews 93, 961–992. 10.1152/physrev.00010.2012. 23899559

[56] Buzsáki G. (2006). Rhythms of the Brain (Oxford University Press) 10.1093/acprof:oso/9780195301069.001.0001.

[57] Watrous A.J., Fried I., and Ekstrom A.D. (2011). Behavioral correlates of human hippocampal delta and theta oscillations during navigation. Journal of Neurophysiology 105, 1747–1755. 10.1152/jn.00921.2010. 21289136

[58] Schultheiss N.W., Schlecht M., Jayachandran M., Brooks D.R., McGlothan J.L., Guilarte T.R., and Allen T.A. (2020). Awake Delta and Theta-Rhythmic Hippocampal Network Modes During Intermittent Locomotor Behaviors in the Rat. Behavioral Neuroscience 134, 529–546. 32672989 10.1037/bne0000409 PMC8193833

[59] Scheffer L.K., Xu C.S., Januszewski M., Lu Z., Takemura S., Hayworth K.J., Huang G.B., Shinomiya K., Maitlin-Shepard J., Berg S., (2020). A connectome and analysis of the adult *Drosophila* central brain. eLife 9, e57443. 10.7554/eLife.57443. 32880371 PMC7546738

[60] Pfeiffer B.D., Ngo T.-T.B., Hibbard K.L., Murphy C., Jenett A., Truman J.W., and Rubin G.M. (2010). Refinement of Tools for Targeted Gene Expression in *Drosophila*. Genetics 186, 735–755. 10.1534/genetics.110.119917. 20697123 PMC2942869

[61] Jenett A., Rubin G.M., Ngo T.-T.B., Shepherd D., Murphy C., Dionne H., Pfeiffer B.D., Cavallaro A., Hall D., Jeter J., (2012). A GAL4-Driver Line Resource for *Drosophila* Neurobiology. Cell Reports 2, 991–1001. 10.1016/j.celrep.2012.09.011. 23063364 PMC3515021

[62] Kvon E.Z., Kazmar T., Stampfel G., Yáñez-Cuna J.O., Pagani M., Schernhuber K., Dickson B.J., and Stark A. (2014). Genome-scale functional characterization of *Drosophila* developmental enhancers in vivo. Nature 512, 91–95. 10.1038/nature13395. 24896182

[63] Tirian L., and Dickson B.J. (2017). The VT GAL4, LexA, and split-GAL4 driver line collections for targeted expression in the Drosophila nervous system (Neuroscience) 10.1101/198648.

[64] Pfeiffer B.D., Jenett A., Hammonds A.S., Ngo T.-T.B., Misra S., Murphy C., Scully A., Carlson J.W., Wan K.H., Laverty T.R., (2008). Tools for neuroanatomy and neurogenetics in *Drosophila*. Proc. Natl. Acad. Sci. U.S.A. 105, 9715–9720. 10.1073/pnas.0803697105. 18621688 PMC2447866

[65] Dana H., Mohar B., Sun Y., Narayan S., Gordus A., Hasseman J.P., Tsegaye G., Holt G.T., Hu A., Walpita D., (2016). Sensitive red protein calcium indicators for imaging neural activity. eLife 5, e12727. 10.7554/eLife.12727. 27011354 PMC4846379

[66] Dana H., Sun Y., Mohar B., Hulse B.K., Kerlin A.M., Hasseman J.P., Tsegaye G., Tsang A., Wong A., Patel R., (2019). High-performance calcium sensors for imaging activity in neuronal populations and microcompartments. Nat Methods 16, 649–657. 10.1038/s41592-019-0435-6. 31209382

[67] Wolff T., and Rubin G.M. (2018). Neuroarchitecture of the *Drosophila* central complex: A catalog of nodulus and asymmetrical body neurons and a revision of the protocerebral bridge catalog. J Comp Neurol 526, 2585–2611. 10.1002/cne.24512. 30084503 PMC6283239

[68] Meissner G.W., Nern A., Dorman Z., DePasquale G.M., Forster K., Gibney T., Hausenfluck J.H., He Y., Iyer N.A., Jeter J., (2023). A searchable image resource of *Drosophila* GAL4 driver expression patterns with single neuron resolution. eLife 12, e80660. 10.7554/eLife.80660. 36820523 PMC10030108

[69] Zhang Y., Rózsa M., Bushey D., Zheng J., Reep D., Broussard G.J., Tsang A., Tsegaye G., Patel R., Narayan S., (2020). jGCaMP8 Fast Genetically Encoded Calcium Indicators.

[70] Wang L., Wu C., Peng W., Zhou Z., Zeng J., Li X., Yang Y., Yu S., Zou Y., Huang M., (2022). A high-performance genetically encoded fluorescent indicator for in vivo cAMP imaging. Nat Commun 13, 5363. 10.1038/s41467-022-32994-7. 36097007 PMC9468011

[71] Vijayan V., Wang F., Wang K., Chakravorty A., Adachi A., Akhlaghpour H., Dickson B.J., and Maimon G. (2023). A rise-to-threshold process for a relative-value decision. Nature 619, 563–571. 10.1038/s41586-023-06271-6. 37407812 PMC10356611

[72] Thornquist S.C., Langer K., Zhang S.X., Rogulja D., and Crickmore M.A. (2020). CaMKII Measures the Passage of Time to Coordinate Behavior and Motivational State. Neuron 105, 334–345.e9. 10.1016/j.neuron.2019.10.018. 31786014 PMC7374950

[73] Maimon G., Straw A.D., and Dickinson M.H. (2010). Active flight increases the gain of visual motion processing in *Drosophila*. Nat Neurosci 13, 393–399. 10.1038/nn.2492. 20154683

[74] Kim A.J., Fenk L.M., Lyu C., and Maimon G. (2017). Quantitative Predictions Orchestrate Visual Signaling in *Drosophila*. Cell 168, 280–294.e12. 10.1016/j.cell.2016.12.005. 28065412 PMC6320683

[75] Wilson R.I., and Laurent G. (2005). Role of GABAergic Inhibition in Shaping Odor-Evoked Spatiotemporal Patterns in the *Drosophila* Antennal Lobe. J. Neurosci. 25, 9069–9079. 10.1523/JNEUROSCI.2070-05.2005. 16207866 PMC6725763

[76] Kim S.S., Hermundstad A.M., Romani S., Abbott L.F., and Jayaraman V. (2019). Generation of stable heading representations in diverse visual scenes. Nature 576, 126–131. 10.1038/s41586-019-1767-1. 31748750 PMC8115876

[77] Reiser M.B., and Dickinson M.H. (2008). A modular display system for insect behavioral neuroscience. Journal of Neuroscience Methods 167, 127–139. 10.1016/j.jneumeth.2007.07.019. 17854905

[78] Green J., Adachi A., Shah K.K., Hirokawa J.D., Magani P.S., and Maimon G. (2017). A neural circuit architecture for angular integration in *Drosophila*. Nature 546, 101–106. 10.1038/nature22343. 28538731 PMC6320684

[79] Moore R.J.D., Taylor G.J., Paulk A.C., Pearson T., Van Swinderen B., and Srinivasan M.V. (2014). FicTrac: A visual method for tracking spherical motion and generating fictive animal paths. Journal of Neuroscience Methods 225, 106–119. 10.1016/j.jneumeth.2014.01.010. 24491637

[80] Handler A., Graham T.G.W., Cohn R., Morantte I., Siliciano A.F., Zeng J., Li Y., and Ruta V. (2019). Distinct Dopamine Receptor Pathways Underlie the Temporal Sensitivity of Associative Learning. Cell 178, 60–75.e19. 10.1016/j.cell.2019.05.040. 31230716 PMC9012144

[81] Giovannucci A., Friedrich J., Gunn P., Kalfon J., Brown B.L., Koay S.A., Taxidis J., Najafi F., Gauthier J.L., Zhou P., (2019). CaImAn an open source tool for scalable calcium imaging data analysis. eLife 8, e38173. 10.7554/eLife.38173. 30652683 PMC6342523

[82] Wolff T., Iyer N.A., and Rubin G.M. (2015). Neuroarchitecture and neuroanatomy of the *Drosophila* central complex: A GAL4-based dissection of protocerebral bridge neurons and circuits. J. Comp. Neurol 523, 997–1037. 10.1002/cne.23705. 25380328 PMC4407839

[83] Turner-Evans D.B., Jensen K.T., Ali S., Paterson T., Sheridan A., Ray R.P., Wolff T., Lauritzen J.S., Rubin G.M., Bock D.D., (2020). The Neuroanatomical Ultrastructure and Function of a Biological Ring Attractor. Neuron 108, 145–163.e10. 10.1016/j.neuron.2020.08.006. 32916090 PMC8356802

[84] Edgar R., Domrachev M., and Lash A.E. (2002). Gene Expression Omnibus: NCBI gene expression and hybridization array data repository. Nucleic Acids Research 30, 207–210. 10.1093/nar/30.1.207. 11752295 PMC99122

[85] Barrett T., Wilhite S.E., Ledoux P., Evangelista C., Kim I.F., Tomashevsky M., Marshall K.A., Phillippy K.H., Sherman P.M., Holko M., (2012). NCBI GEO: archive for functional genomics data sets—update. Nucleic Acids Research 41, D991–D995. 10.1093/nar/gks1193. 23193258 PMC3531084

[86] Berg S., and Schlegel P. (2017). neuprint-python. Version 0.4.15.

[87] Fisher N.I., and Lee A.J. (1983). A correlation coefficient for circular data. Biometrika 70, 327–332.

[88] Virtanen P., Gommers R., Oliphant T.E., Haberland M., Reddy T., Cournapeau D., Burovski E., Peterson P., Weckesser W., Bright J., (2020). SciPy 1.0: fundamental algorithms for scientific computing in Python. Nat Methods 17, 261–272. 10.1038/s41592-019-0686-2. 32015543 PMC7056644

[89] Berens P. (2009). CircStat : A MATLAB Toolbox for Circular Statistics. J. Stat. Soft. 31. 10.18637/jss.v031.i10.

