# Supplementary material for: Neuronal calcium spikes enable vector inversion in the Drosophila brain
Source: bioRxiv. 2023 Nov 28:2023.11.24.568537. Preprint. [Version 3] doi: 10.1101/2023.11.24.568537 (PMC10705278; doi:10.1101/2023.11.24.568537)
Supplement: Supplement 1 [file NIHPP2023.11.24.568537V3-supplement-1.pdf]

bioRxiv preprint doi: https://doi.org/10.1101/2023.11.24.568537; this version posted November 28, 2023. The copyright holder for this preprint (which was not certified by peer review) is the author/funder, who has granted bioRxiv a license to display the preprint in perpetuity. It is made available under aCC-BY 4.0 International license.

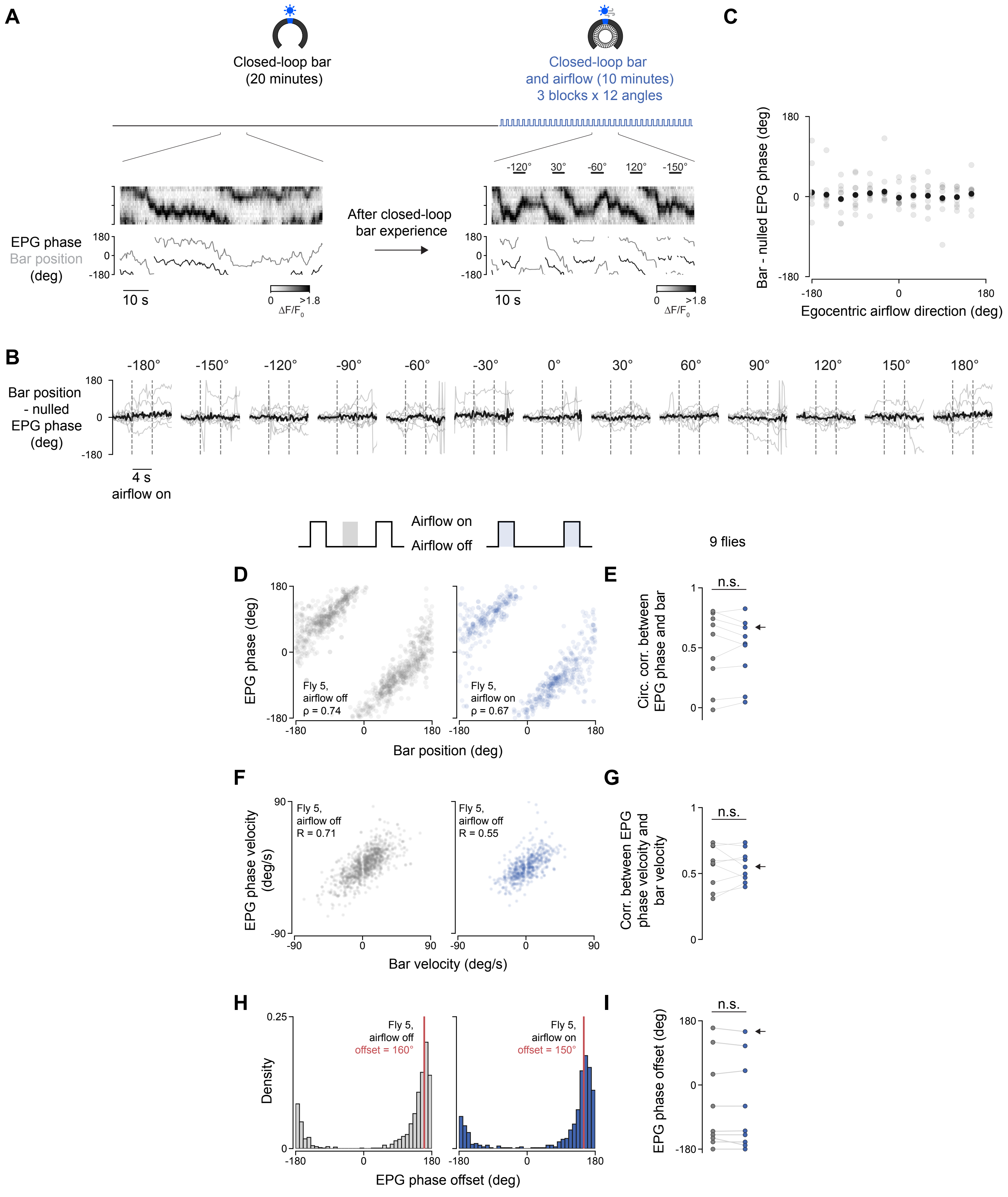

Figure S1. EPG neurons track the fly's heading equally well in the presence and absence of open-loop airflow pulses. See next page for figure legend.

bioRxiv preprint doi: https://doi.org/10.1101/2023.11.24.568537; this version posted November 28, 2023. The copyright holder for this preprint (which was not certified by peer review) is the author/funder, who has granted bioRxiv a license to display the preprint in perpetuity. It is made available under aCC-BY 4.0 International license.

Figure S1. EPG neurons track the fly's heading equally well in the presence and absence of open-loop airflow pulses. (A) Example trace of EPG activity in the ellipsoid body in the closed-loop visual and open-loop airflow experiment. The ellipsoid body is split and unfolded as to display the donut as a single 16- wedge long array over time (gray color mesh), the color corresponds to the intensity of activity along each sector at every imaging frame. The position of the EPG activity peak is the population vector average estimate along the ellipsoid body, called the EPG phase (shown in black lines). The position of the visual stimulus on the LED screen is shown as a gray line. **(B)** Correspondence between the EPG phase and the bar position as a function of the 12 different air puff directions. The arbitrary offset between the EPG phase and bar position was zeroed at the beginning of each airflow trial, to highlight perturbations of the EPG phase-to-bar correspondence during the stimulus period. Average of 9 flies. **(C)** Average values of the last two seconds of airflow stimulation for the data shown in B. 9 flies. **(D)** Correspondence between the EPG phase position to the visual stimulus position on the screen in an example fly. The visual stimulus is never off: in the "airflow off" condition (left panel), only the closed-loop visual stimulus is present. In the "airflow on" condition (right panel), both the closed-loop visual stimulus and the open-loop airflow are present. The circular correlation coefficient ρ is noted. **(E)** Circular correlation between the EPG phase and the bar position for 9 flies, with airflow on and airflow off. Gray dots show the "airflow off" condition, blue dots show the "airflow on" condition, and thin lines pair values for individual flies. The arrow highlights the data points corresponding to the example fly in D. **(F)** EPG phase velocity as a function of the visual stimulus velocity in an example fly. The unity line is shown in dotted black. Pearson's R is noted. **(G)** Correlation between the EPG phase velocity and the visual stimulus velocity in 9 flies. Gray dots show the "airflow off" condition, blue dots show the "airflow on" condition, and thin lines pair values for individual flies. The arrow highlights the data points corresponding to the example fly in F. **(H)** Quantification of the arbitrary offset between the EPG phase and the visual stimulus for an example fly. The circular difference between the EPG phase and the bar position on the screen was divided into 36 bins of 10°, and the bin with the highest counts (labeled by the red line) was selected as the offset for the airflow on and airflow off conditions for each fly. **(I)** Summary of the arbitrary offset between the EPG phase and the bar position on the screen for 9 flies. Each point in this plot represents the mean difference between the offset-nulled EPG phase and the bar position over the last 2 s of the airflow stimulus. Gray dots show the "airflow off" condition, blue dots show the "airflow on" condition, and thin lines pair values for individual flies. The arrow highlights the data points corresponding to the example fly in H.

bioRxiv preprint doi: https://doi.org/10.1101/2023.11.24.568537; this version posted November 28, 2023. The copyright holder for this preprint (which was not certified by peer review) is the author/funder, who has granted bioRxiv a license to display the preprint in perpetuity. It is made available under aCC-BY 4.0 International license.

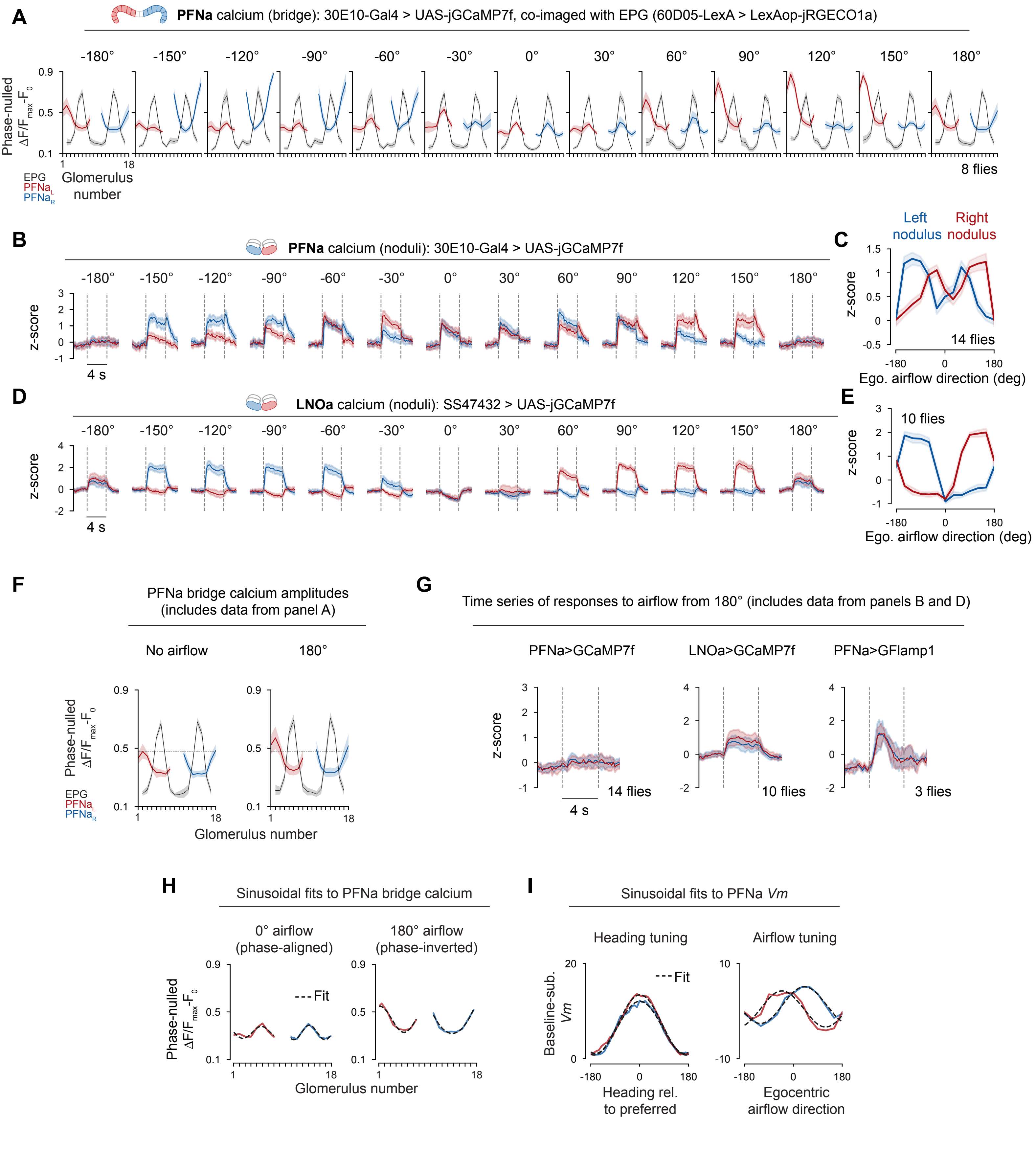

Figure S2. Tuning curves to airflow stimuli in the PFNa system. See next page for figure legend.

bioRxiv preprint doi: https://doi.org/10.1101/2023.11.24.568537; this version posted November 28, 2023. The copyright holder for this preprint (which was not certified by peer review) is the author/funder, who has granted bioRxiv a license to display the preprint in perpetuity. It is made available under aCC-BY 4.0 International license.

Figure S2. Tuning curves to airflow stimuli in the PFNa system. (A) Average data for the PFNa neurons' phase alignment (red and blue traces) relative to the EPG heading neurons (black trace); we show the full series of airflow angles tested. We nulled the EPG phases on both sides of the bridge independently and at every imaging frame, corresponding to one time point. The EPG phase estimate at each time point was aligned to the middle glomeruli in a virtual protocerebral bridge. We then shifted the PFNa phase using the EPG phase estimate to determine its peak relative to the EPG neurons' phase. **(B)** Time course of mean PFNa population activity, as measured via calcium signals, in the noduli as a function of airflow direction. The left nodulus is shown in blue, and the right nodulus is shown in red. The egocentric airflow direction is shown above each panel, and the time of airflow stimulus presentation is flanked by the dashed gray lines. **(C)** Tuning curve of PFNa-derived calcium signals in the noduli to egocentric airflow direction. Each point in this plot represents the mean value of the noduli z-score over the last 2 s of the airflow stimulus presentation in B. Note that the tuning curves for PFNa population activity in the noduli are double-phased. **(D)** Time course of mean LNOa population activity in the noduli as a function of airflow direction. The LNOa neurons are one of the numerically dominant synaptic inputs to the PFNa neurons in the noduli. The left nodulus is shown in blue, and the right nodulus is shown in red. The egocentric airflow direction is shown above each panel, and the time of airflow stimulus presentation is flanked by the dashed gray lines. **(E)** Tuning curve of PFNa-derived calcium signals in the noduli to egocentric airflow direction. Each point in this plot represents the mean value of the noduli z-score over the last 2 s of the airflow stimulus presentation in D. Note that the tuning curves for LNOa calcium have a single peak. **(F)** Comparison of the phase-nulled calcium signals of PFNa neurons in the absence of airflow (left panel) and when airflow is blown from directly behind the fly (180°, right panel). The horizontal dotted line marks the highest mean calcium responses in the no airflow condition, to aid comparison of the values in the two panels; note that both sinusoids peak at values above the line in the 180° airflow condition. **(G)** Reproduction of data from panels B and D (left two panels showing GCaMP signals), plus GFlamp1[70] signals in PFNa neurons measured at the noduli to in response to airflow presented from the egocentric rearmost angle (180°). GFlamp1 is a cAMP reporter, but displays non-negligible cGMP binding as well[70]. Note that whereas PFNa calcium shows minimal change during the stimulus presentation, the LNOa neurons are responsive. In addition, GFlamp1 signal rises within the PFNa neurons themselves shortly after the stimulus onset. **(H)** Sinusoidal function fits to the average phase-nulled PFNa calcium bumps elicited by frontal airflow (left panel) and airflow from the rear (right panel). The fit is shown as a dotted black line; the data is replotted from panel A, using the same color conventions. **(I)** Sinusoidal function fits to the average baseline-subtracted *Vm*, as a function of heading direction and airflow. The fit is shown as a dotted black line; the data is replotted from Figure 3C and 3E. Data from left-bridge-innervating PFNa neurons is shown in red, whereas data from right-bridge-innervating neurons is shown in blue.

bioRxiv preprint doi: https://doi.org/10.1101/2023.11.24.568537; this version posted November 28, 2023. The copyright holder for this preprint (which was not certified by peer review) is the author/funder, who has granted bioRxiv a license to display the preprint in perpetuity. It is made available under aCC-BY 4.0 International license.

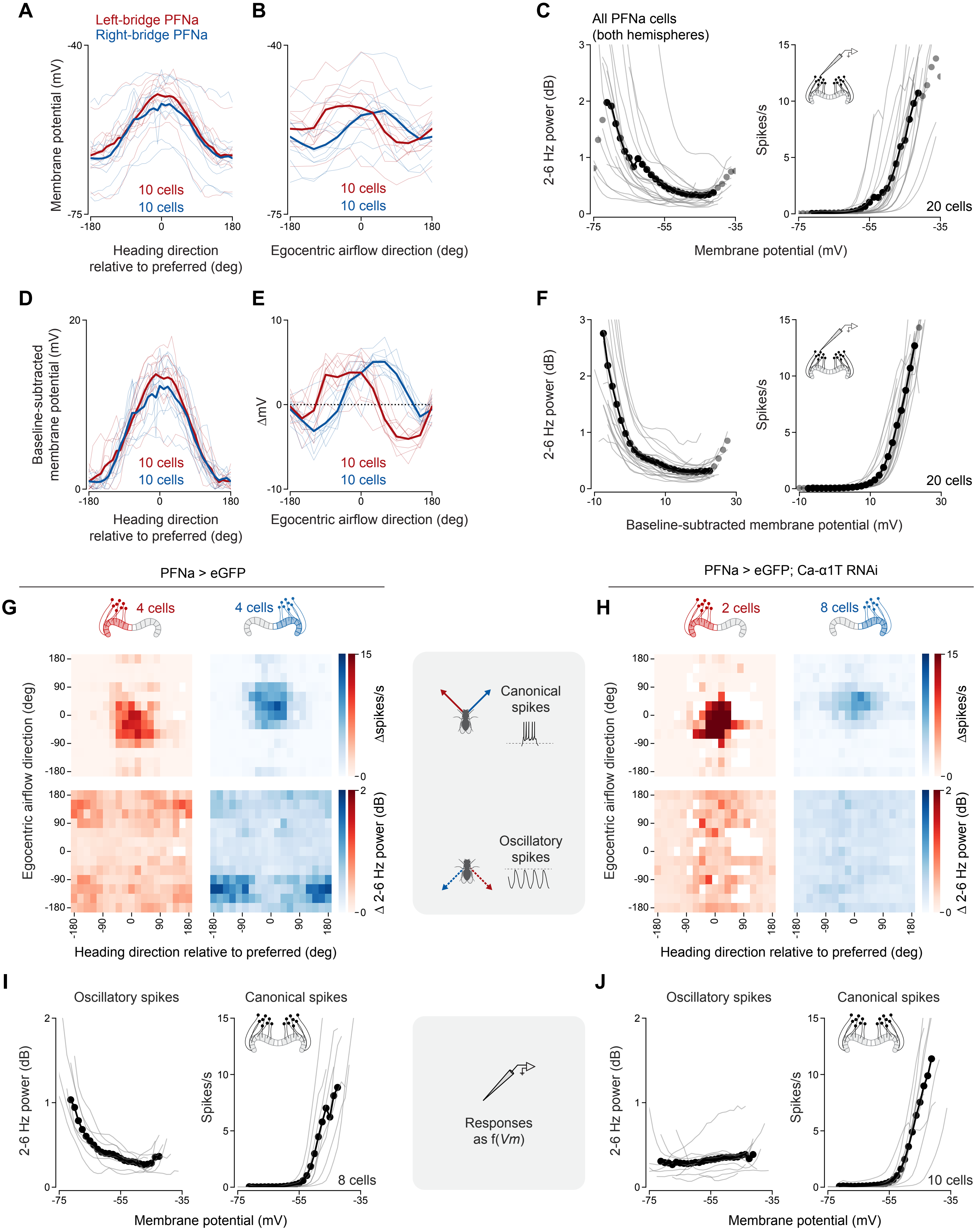

Figure S3. Raw and normalized *Vm* tuning curves from PFNa neurons. See next page for figure legend.

bioRxiv preprint doi: https://doi.org/10.1101/2023.11.24.568537; this version posted November 28, 2023. The copyright holder for this preprint (which was not certified by peer review) is the author/funder, who has granted bioRxiv a license to display the preprint in perpetuity. It is made available under aCC-BY 4.0 International license.

Figure S3. Raw and normalized *Vm* tuning curves from PFNa neurons. (A) Tuning of raw PFNa *Vm* to the fly's heading, as estimated by the angular position of the closed-loop bar on the visual display. All tuning curves have been phase-aligned to have their peak at 0˚ (**Methods**). Left-bridge PFNa neurons: red. Right-bridge PFNa neurons: blue. Thin lines: single fly averages. Thick lines: population averages. Data from 20 cells (10 cells per hemisphere). **(B)** Raw *Vm* airflow direction tuning curves of PFNa neurons. These tuning curves have not been shifted. Thin lines: single fly averages. Thick lines: population averages. Data from 20 cells (10 cells per hemisphere). **(C)** 2-6 Hz power (left panel) and canonical spike rate (right panel) as a function of raw *Vm* in PFNa neurons. Responses of right- and left-bridge PFNa neurons were pooled. Thin lines: single fly averages. Thick lines: population averages. Mean points in which less than 50% of the curves contributed to the bin are displayed in gray. Data from 20 cells. **(D)** Same as in A, but showing the baseline-subtracted *Vm* instead of the raw *Vm*. The baseline was defined as the minimum value of the heading tuning curve (see **Methods**). **(E)** Same as in B, but showing the difference in *Vm* pre- and post-airflow stimulus instead of the raw *Vm*. **(F)** Same as in C, but showing the baseline-subtracted *Vm* instead of the raw *Vm*. The baseline was defined as the minimum value of the heading tuning curve (see **Methods**). Mean points in which less than 50% of the curves contributed to the bin are displayed in gray. **(G)** Conjunctive tuning of left- and right-bridge PFNa cells to the direction of airflow and heading. These recordings were made from flies of the genetic background used to create the TRiP RNAi libraries[16] ("empty RNAi control"), but where eGFP was expressed in PFNa neurons. **(H)** Same as in panel G, but in PFNa cells carrying the construct TRiP.HMS01948, which allows for expression (under UAS control) of a double-stranded RNA that targets Ca-α1T transcripts for degradation (Ca-α1T RNAi). **(I)** Same as in C, but recording from PFNa cells in flies of the empty RNAi control genotype. **(J)** Same as in C, but recording from PFNa cells carrying the construct TRiP.HMS01948 (Ca-α1T RNAi).

bioRxiv preprint doi: https://doi.org/10.1101/2023.11.24.568537; this version posted November 28, 2023. The copyright holder for this preprint (which was not certified by peer review) is the author/funder, who has granted bioRxiv a license to display the preprint in perpetuity. It is made available under aCC-BY 4.0 International license.

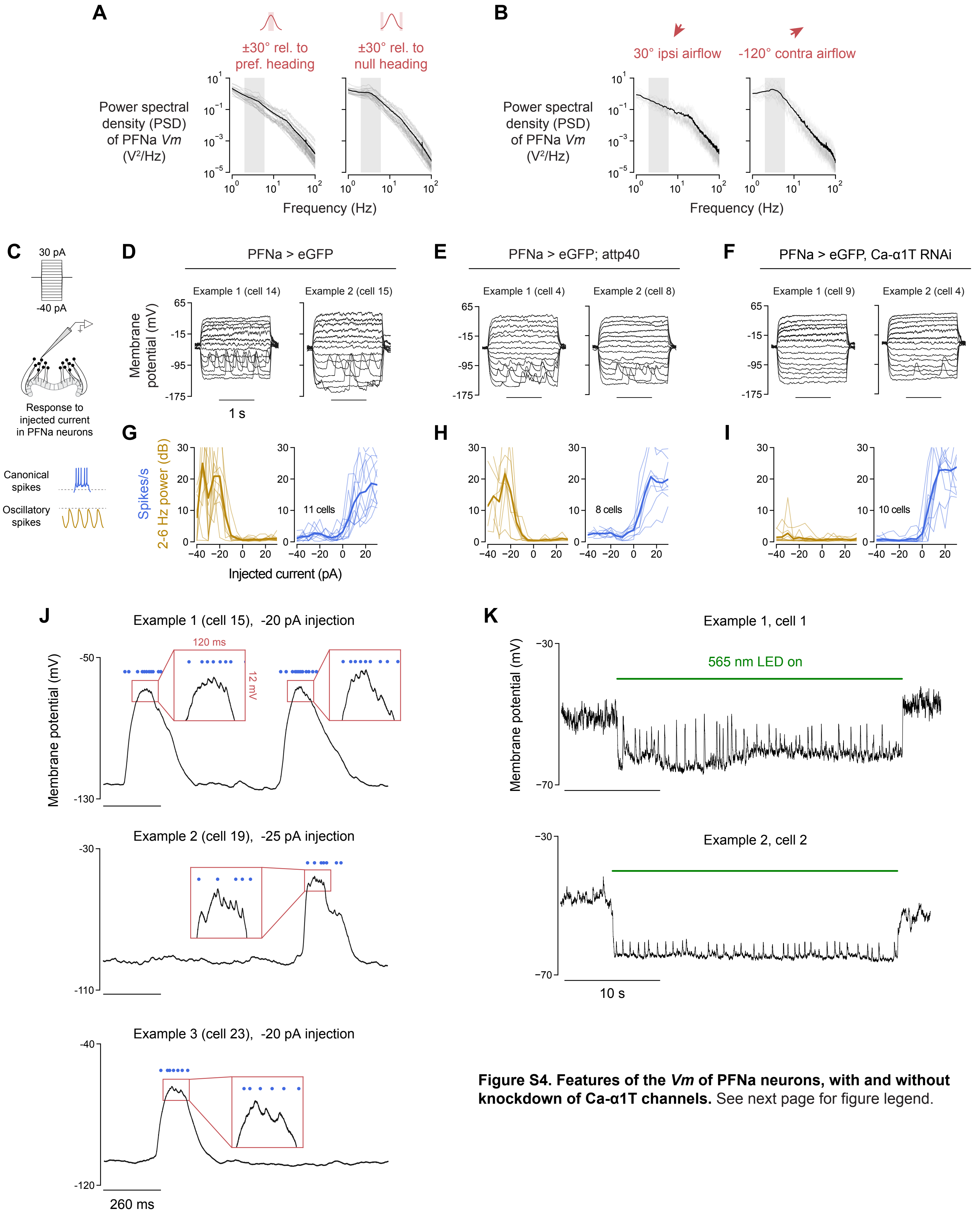

Figure S4. Features of the *Vm* of PFNa neurons, with and without knockdown of Ca-α1T channels. See next page for figure legend.

bioRxiv preprint doi: https://doi.org/10.1101/2023.11.24.568537; this version posted November 28, 2023. The copyright holder for this preprint (which was not certified by peer review) is the author/funder, who has granted bioRxiv a license to display the preprint in perpetuity. It is made available under aCC-BY 4.0 International license.

Figure S4. Features of the *Vm* of PFNa neurons, with and without knockdown of Ca-α1T channels. (A) Power spectral density (PSD) of the *Vm* of PFNa neurons during moments where the fly's heading was aligned to the cell's preferred direction (left panel) and moments where it was aligned to the null direction (right panel). Each panel shows a 60° bin of heading data (e.g. the left panel shows the PSD for the *Vm* of PFNa neurons when the fly's heading was within ±30° relative to each cell's preferred heading). The gray box indicates the 2-6 Hz band. Values from 20 cells. **(B)** Same as in A, but for depolarizing stimuli (airflow from 30° for right-bridge-innervating PFNa neurons and -30° for left-bridge-innervating PFNa neurons) and hyperpolarizing stimuli (airflow from -120° for right-bridge-innervating PFNa neurons and 120° for left-bridge-innervating PFNa neurons). Values from 20 cells. **(C)** Schematic of the current injection experiment. Each experiment consisted of a family of 15 current steps spaced 5 pA apart, starting at -40 pA and ending at 30 pA. We pooled cells innervating both brain hemispheres in this figure. **(D)** Representative families of current injections for 2 out of 11 different PFNa cells. This genotype is SS02255>UAS-2xeGFP, where no intentional perturbations were inserted. To the extent that it was possible, the recordings were preferentially acquired while the fly was quiescent. This was done to minimize depolarizing input from self-motion or membrane potential changes due to the visual stimulus entering or exiting the cell's receptive field. **(E)** Same as in D, but in empty RNAi control genotype flies. **(F)** Same as in E, but in PFNa neurons expressing TRiP.HMS01948, which targets the Ca-α1T transcript (Ca-α1T RNAi). **(G)** 2-6 Hz power (left panel, dark yellow) and canonical spike responses (right panel, blue) as a function of membrane potential in PFNa neurons in SS02255>UAS-2xeGFP flies, where no intentional perturbations were inserted. The responses of right- and left-bridge innervating neurons were pooled. Thin lines represent individual cells, thick lines represent the average responses of 11 cells. **(H)** Same as in G, but in empty RNAi control genotype flies. 8 cells. **(I)** Same as in (H), but in PFNa cells expressing 2xeGFP and TRiP.HMS01948, which targets the Ca-α1T transcript. 10 cells. **(J)** Three example traces of current injection steps that yielded sodium spiking at the peak of a calcium spike. The red insets are magnified epochs highlighting the sodium spikes (blue dots). (Note that the cell bodies of *Drosophila* neurons connect to the cell arbors through a thin neurite that considerably filters voltage signals from the processes. Spikes in the PFNa neuron recordings are hard to visualize in raw voltage traces but are easily detected by filtering and thresholding the membrane potential.) **(K)** Two example traces of PFNa neurons showing calcium spiking in response to GtACR1-mediated hyperpolarization. We stimulated the fly using a 565 nm LED. The light intensity was measured to be 21 μW/mm2 prior to inserting a neutral density filter in the light path to reduce the magnitude of optogenetic stimulation.

bioRxiv preprint doi: https://doi.org/10.1101/2023.11.24.568537; this version posted November 28, 2023. The copyright holder for this preprint (which was not certified by peer review) is the author/funder, who has granted bioRxiv a license to display the preprint in perpetuity. It is made available under aCC-BY 4.0 International license.

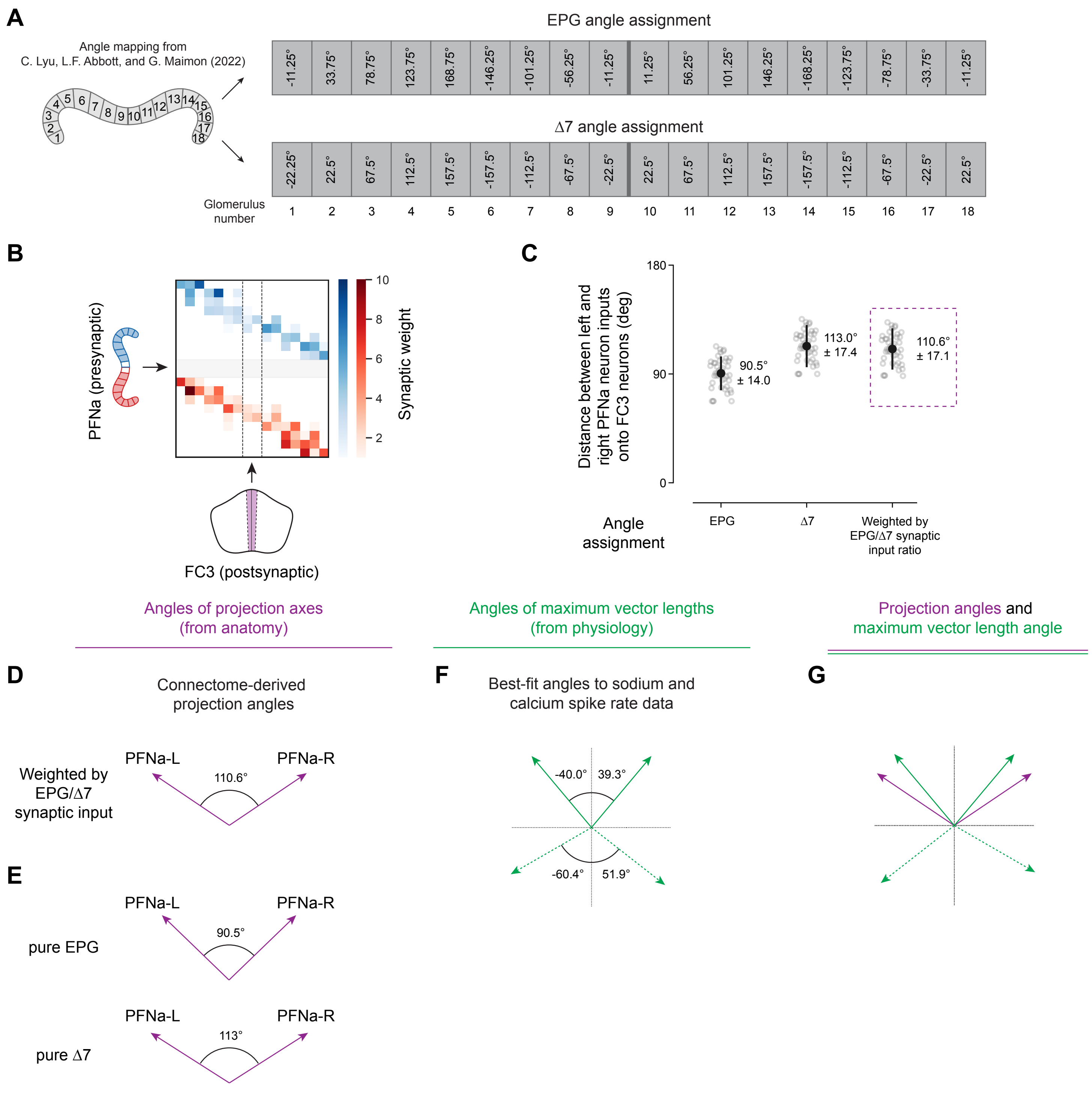

Figure S5. Analysis of the vector-axis directions and peak-vector length angles in PFNa neurons. (A) Angular indexing of the protocerebral bridge, with different schemes for the EPG neurons and the Δ7 neurons. These angles were experimentally defined in Lyu et al 2022[1] and we adopt them in this study. Adjacent glomeruli are generally spaced 45° apart in both cell types, but the indexing values of the Δ7 neurons are shifted to those from the EPG neurons by 1/4th of a glomerulus (11.25°). Note that the EPG neurons do not innervate the outermost two glomeruli (1 and 18); these angles were inferred given the bridge innervation pattern of the PEN (and PFN) neurons[78]. **(B)** Connectivity matrix of the PFNa neurons onto the FC3 neurons, averaged over neuron instances that innervate the same bridge glomerulus or fan-shaped body column. Every fan-shaped body column innervated by the FC3 neurons receives input from both the left- and the right-bridge PFNa neurons. **(C)** Angle difference between left-bridge and right-bridge PFNa neuron inputs onto the FC3 neurons. Each circle corresponds to the values calculated for individual FC3 neurons, using the bridge indexing of either EPG alone, Δ7 alone, or a weighted combination. **(D)** Anatomically defined angle between the left and right PFNa vectors. 110.6° corresponds to the value calculated in C via connectome analysis. **(E)** Same as in D, but assuming pure EPG or pure Δ7 input. **(F)** We fit the heading-and-airflow 2-D heatmaps for spiking responses using the quadratic model in **Figure 5**. The best-fit angles for the preferred airflow directions are shown as the angles for each of the four vectors. **(G)** Overlay of the angles of the projection axes (purple) and the angles of the maximum vector lengths (green) as estimated in panels D and F.

bioRxiv preprint doi: https://doi.org/10.1101/2023.11.24.568537; this version posted November 28, 2023. The copyright holder for this preprint (which was not certified by peer review) is the author/funder, who has granted bioRxiv a license to display the preprint in perpetuity. It is made available under aCC-BY 4.0 International license.

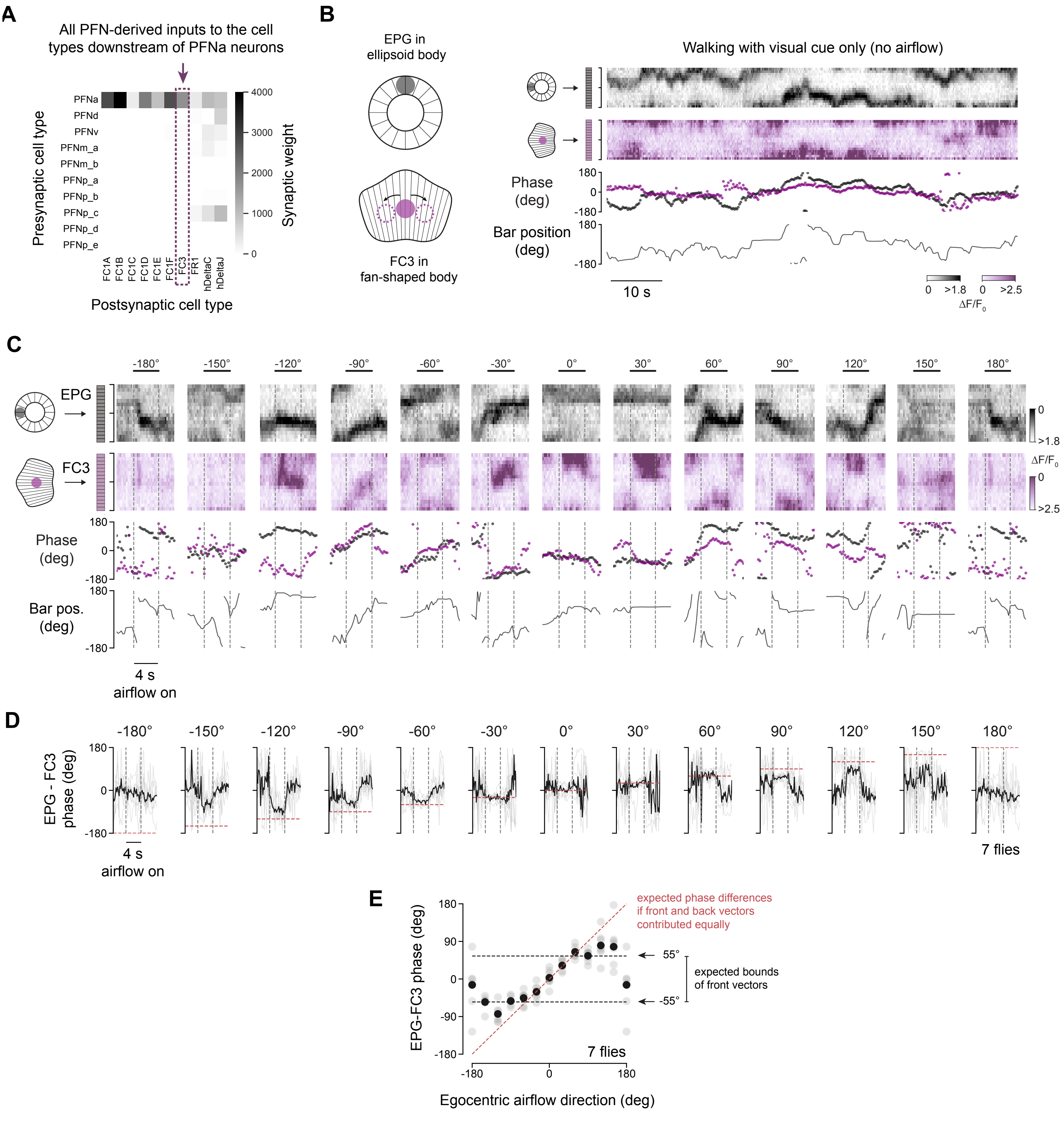

Figure S6. Assessment of the ability of FC3 neurons to signal the allocentric direction of air puffs. See next page for figure legend.

bioRxiv preprint doi: https://doi.org/10.1101/2023.11.24.568537; this version posted November 28, 2023. The copyright holder for this preprint (which was not certified by peer review) is the author/funder, who has granted bioRxiv a license to display the preprint in perpetuity. It is made available under aCC-BY 4.0 International license.

Figure S6. Assessment of the ability of FC3 neurons to signal the allocentric direction of air puffs. (A) Connectivity matrix showing all of the identified PFN neurons in the Drosophila hemibrain connectome[59], and how they output onto the 10 types of columnar cell classes postsynaptic to PFNa neurons: the FC1A, FC1B, FC1C, FC1D, FC1E, FC1F, FC3, FR1, hΔC, and hΔJ cell classes[4]. The FR1, hΔC, and hΔJ neurons receive synaptic input from additional types of PFN cells, potentially introducing confounds into our analysis of the PFNa neurons' synaptic output; we thus chose not to study these cell classes. The FC1 and FC3 neurons, in contrast, receive essentially all of their PFN-related synaptic input from PFNa neurons. The FC1 cells are the largest recipients of monosynaptic PFNa neuron input, but the six subtypes (FC1A-E) are challenging to parse using light microscopy. Therefore, we focused on the FC3 neurons, which have an identifiable anatomy, as our model postsynaptic neuron class. The FC3 neurons also receive heavy synaptic input from the FC1 neurons (not shown in this graph). **(B)** Example trace of an experiment where we simultaneously imaged the EPG and FC3 neurons. The EPG bump was imaged in the ellipsoid body, whereas the FC3 were imaged in the fan-shaped body, pictured here is the FC3 activity in layer 5 of the FB. The phase of the two population signals is overlaid in the third row, and the position of the visual stimulus on the screen is shown on the fourth row. **(C)** Example airflow responses of the EPG and FC3 neurons at the 12 airflow angles tested. The airflow-on period is flanked by the dotted lines, and the content and structure of the panels is otherwise the same as in B. **(D)** Time course of the difference of the FC3 phase position relative to the EPG phase during periods of airflow stimulation. The airflow-on period is flanked by the dotted lines, and the red dotted line denotes the expected magnitude of the phase difference if the PFNa system were operating as a four-vector set with equal amplitudes. The thin lines show individual flies, the thick lines show the average values across 7 flies. **(E)** Difference of the FC3 phase position relative to the EPG phase in the context of airflow stimulation like the one shown in D. The values shown here correspond to the average phase difference over the last two seconds of the airflow stimulus. The gray dots show individual flies, the black dots show the average values across 7 flies. The anatomically-defined expected bounds of the front vectors (see **Supplementary Text** and **Figure S5**) are noted and marked with dotted black lines; the expected phase differences if the front and back vectors contributed equally are noted in red.

bioRxiv preprint doi: https://doi.org/10.1101/2023.11.24.568537; this version posted November 28, 2023. The copyright holder for this preprint (which was not certified by peer review) is the author/funder, who has granted bioRxiv a license to display the preprint in perpetuity. It is made available under aCC-BY 4.0 International license.

Supplementary Text

When we fit our model to *Vm*, spiking, and oscillation data, we used fixed angle offsets of ±45° for the airflow tuning curves. However, we also fit these data allowing these offset angles to be free parameters. When we did this for the *Vm* data, we obtained angles of -41.9° and 41.3°. Although these angles differ from the ±45° assumed in our model, the improvement in the fits obtained by introducing these extra free parameters was small, only 0.02% additional variance explained for the left-bridge and right-bridge PFNa neurons. Allowing the angles to be free parameters for fitting the spiking and oscillation data gave angles of -40.0°, 39.3°, -60.4°, and -51.9° (**Figure S5F**) for the left-bridge and right-bridge spiking data and left-bridge and right-bridge oscillation data, respectively. This allowed the fits to explain an additional 0.8%, 1.0%, 7.3% and 1.2% of the variance in the data. These angles are close to, but slightly offset from, the ±45° expected from a perfectly orthogonal system.

The deviations from orthogonality suggested by the above analysis make quantitative predictions about the nature of heading tuning in the PFNa neurons if a vector sum process aims to accurately signal the allocentric airflow direction. For example, if a PFNa cell were to express its maximal airflow response at +40˚—i.e., 5˚closer to the fly's midline than the +45˚ orthogonal prediction—its response to heading in the fan-shaped body should be shifted by +50˚ (relative to its response in the protocerebral bridge), i.e., 5˚ further from the midline, for the vector sum process to be accurate. Put another way, if a PFNa-encoded vector expresses its maximal vector length when airflow arrives from +40˚, then the axis that this vector points along should be +50˚ for the vector sum process to be accurate. With non-orthogonal axes for a pair of basis vectors, the angle of each axis and the airflow angle at which each basis vector has its maximal projection to that axis are no longer the same angle.

To determine the angular shift in the PFNa projection from the bridge to the fan-shaped body—which determines the axis along which each PFNa vector points—we used the hemibrain connectome[59]. In the bridge, we assigned an angle to each synapse that a PFNa cell receives from an EPG or Δ7 cell, based on standard assumptions of how heading is signaled in the EPG/Δ7 system[1,3] (**Methods, Figure S5A**). We averaged all the synaptic angles onto a given PFNa cell to assign each PFNa cell an overall heading angle for which it codes. We then analyzed the pattern of PFNa synapses onto a specific recipient cell class in the fan-shaped body, FC3 neurons. We found that the mean angular deviation between the left- and right-bridge PFNa cells that co-innervate FC3 cells in a given fan-shaped body column is 90.5° if EPG cells were considered to be the only drivers of PFNa activity and 113° if Δ7 cells were considered to be the only drivers (**Figures S5A, S5B, S5C, and S5E**). Because EPG and Δ7 cells express 11% and 89% of the synapses onto PFNa cells, respectively, their circular weighted average predicts a deviation of 110.6˚ between the left and right PFNa vectors as

bioRxiv preprint doi: https://doi.org/10.1101/2023.11.24.568537; this version posted November 28, 2023. The copyright holder for this preprint (which was not certified by peer review) is the author/funder, who has granted bioRxiv a license to display the preprint in perpetuity. It is made available under aCC-BY 4.0 International license.

they influence a given FC3 column (**Figures S5C and S5D**). This anatomical calculation thus argues that the projection axes for the two PFNa vectors are offset by 55.4° to the left and right of the midline (**Figure S5D**). As mentioned, the fact that this angle is bigger than 45° is expected given that the peak vector lengths, assessed with physiology, were measured to be closer to the midline than 45˚. Specifically, our physiological measurements of 39˚ and 40˚ predicted projection axes of ±50-51˚, which are in reasonable alignment with the 55˚ estimate extracted from the connectome. The fact that the FC3 phase deviated by no more than ±55˚ from the EPG phase in response to air puffs (**Figure S6E**) is consistent with the possibility of the anatomical projection axes of the two PFNa vectors being separated by ~110˚, as the connectome analysis suggests. This offset angle would limit the maximal deviation of any downstream bump that sums the two front vectors.

Together, our angular measurements across the anatomy and physiology of the PFNa neurons suggest that its vector system could function in a non-orthogonal manner (**Figure S5G**). However, given the rather modest improvements provided by the fitted angular variables mentioned in the first paragraph above, we chose to use the canonical angles of ±45° for our model.
